# Stable and Water-Tolerant Deep Eutectic Solvent from Biomass-Derived 5‑Hydroxymethylfurfural (HMF) and Levulinic Acid

**DOI:** 10.1021/acssuschemeng.5c13551

**Published:** 2026-02-12

**Authors:** Grazia Isa C. Righetti, Sara Rozas, Maria Enrica Di Pietro, Sara Santamaría, Sahar Nasrallah, Mirjana Minceva, Francesco Briatico Vangosa, Santiago Aparicio, Andrea Mele

**Affiliations:** † Department of Chemistry, Materials and Chemical Engineering “G. Natta”, 18981 Politecnico di Milano, 20133 Milano, Italy; ‡ Department of Chemistry, University of Burgos, 09001 Burgos, Spain; § Centre for Cooperative Research on Alternative Energies (CIC energiGUNE), Basque Research and Technology Alliance (BRTA), Álava Technology Park, Albert Einstein 48, 01510 Vitoria-Gasteiz, Spain; ∥ Biothermodynamics, TUM School of Life Sciences, 9184 Technical University of Munich, 85354 Freising, Germany; ⊥ International Research Centre in Critical Raw Materials-ICCRAM, University of Burgos, 09001 Burgos, Spain

**Keywords:** Hydroxymethylfurfural, HMF, levulinic acid, DES, phase-diagram, molecular dynamics, solid−liquid equilibrium, NMR spectroscopy, sustainable chemistry

## Abstract

The development of greener extraction systems is essential to improving the sustainability of biomass valorization processes. Here we report that 5-hydroxymethylfurfural (HMF) and levulinic acid (LEV)—two bioderived platform molecules often coproduced in biomass processing—spontaneously form a stable liquid phase over a broad compositional range. Differential scanning calorimetry (DSC) and thermodynamic modeling reveal a eutectic composition near *x*_HMF_ = 0.39 with a melting temperature of 258 K and significant negative deviation from ideal behavior. The mixture remains liquid across a wide compositional range even in the presence of low water content (0.3–1.0 wt %), exhibiting deep eutectic solvent (DES)-like behavior. Structural and dynamic analyses using DSC, NMR spectroscopy, molecular dynamics (MD) and density functional theory (DFT) calculations uncover a nonideal mixing regime driven by the network of HMF-LEV hydrogen bonding. Notably, water does not disrupt the HMF-LEV network but enhances its structuration at the eutectic composition by moderating LEV-LEV interactions and enabling more favorable HMF-LEV hydrogen bonding. This work shows that the liquid HMF-LEV phase can be generated *in situ* within a heterogeneous biomass surrogate, enabling substantially higher HMF extraction efficiencies than conventional solvents and eliminating the need for additional organic extractants. This provides a proof of concept for a solvent-minimized route to HMF recovery, indicating the HMF-LEV system as a promising, bioderived liquid platform with potential for integration into future resource-efficient biorefinery schemes.

## Graphical abstract

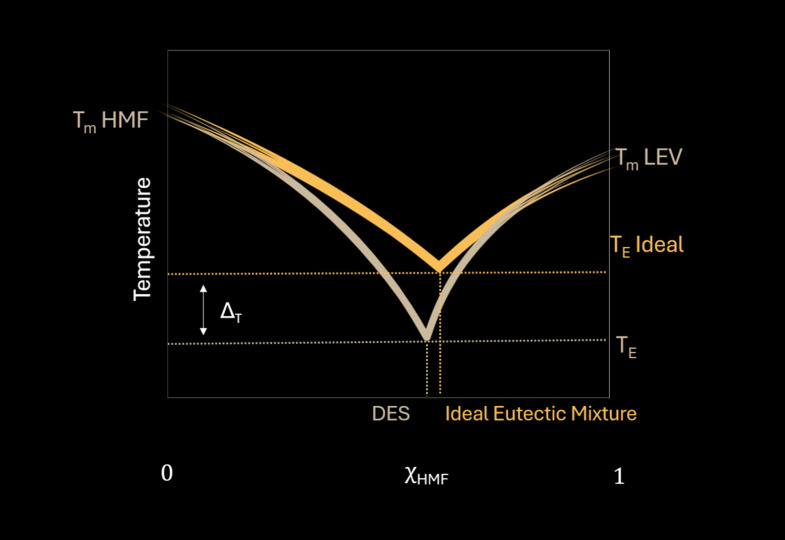

## Introduction

The development of sustainable chemical processes is essential to address environmental challenges^1^ and align with the United Nations Sustainable Development Goals.^2^ Under this perspective, the selective extraction of biobased platform chemicals from reaction media is a critical challenge in biomass valorization workflows.^3^ Among all, 5-hydroxymethylfurfural (HMF) plays a pivotal role in replacing fossil-based intermediates and has attracted extensive interest due to its peculiar reactivity —made possible by the presence of both aldehyde and hydroxymethyl functional groups—making it a key intermediate for the synthesis of high-value chemicals, polymers, and fuels.^4–6^ Despite its promise, its industrial-scale production remains limited by high purification costs and chemical instability,^7,8^ especially in aqueous environments.^9^

Traditional strategies for HMF recovery rely on organic solvents such as toluene^10^ or chloroform,^11^ which are often toxic, volatile, and environmentally burdensome.^12^ Greener approaches—such as biphasic systems, ionic liquids, or deep eutectic solvents (DESs) and mechanochemical processes—have been explored, but they typically require additional components, complex formulations, or fail to prevent degradation pathways.^13–16^

In this work, we report the discovery of a previously unreported binary low-melting mixture composed exclusively of HMF and levulinic acid (LEV), two biobased chemicals derived directly from carbohydrate dehydration (Figure 1).^3^ This eutectic system is stable across a wide compositional range and remains liquid at room temperature, even in the presence of water. Importantly, this binary system enables *in situ* generation of a liquid extraction phase during biomass conversion, which eliminates the need for external solvents or additives. Interestingly, the favorable effect of levulinic acid on HMF extraction yield and its stability was previously observed in lignocellulose biomass treatment, although no molecular rationale was provided.^17^ Herein, we propose that the *in situ* formation of a liquid phase due to eutectic formation between HMF and levulinic acid may underlie these observations, offering a structural explanation based on hydrogen-bonded stabilization.

**Figure 1:**
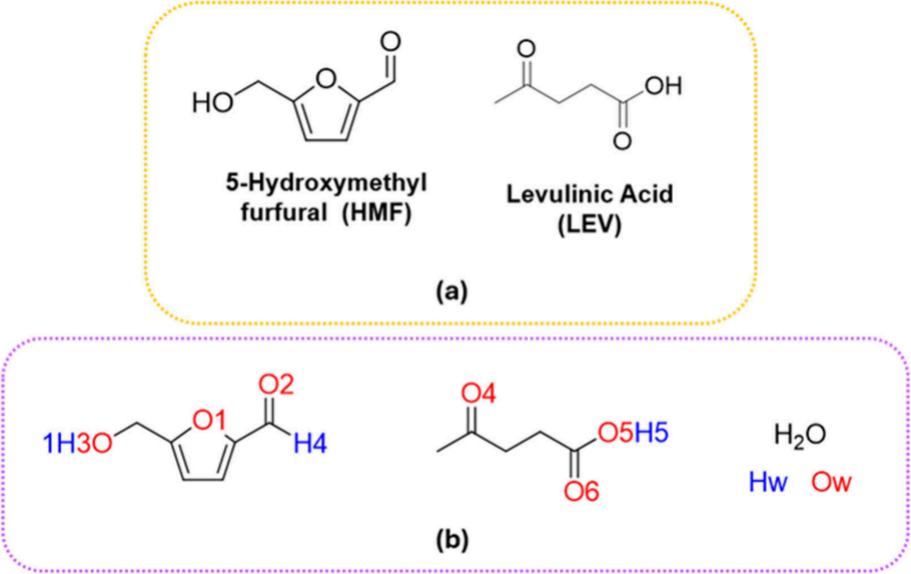
(a) Structures of 5-hydroxymethylfurfural (HMF) and levulinic acid (LEV). (b) Atom labels used in the in-silico study.

LEV, often regarded as an undesirable degradation product of HMF,^18^ is here repurposed as a benign additive (actually, a DES former), reversing its conventional role and contributing to the stabilization of HMF via intermolecular hydrogen bonding. Through combined solid–liquid equilibrium (SLE) measurements, NMR spectroscopy, and computational modeling (DFT and MD), we also demonstrate that small amounts of water not only fail to disrupt the HMF-LEV network but enhance it at eutectic composition by weakening self-association of the pure components.

The discovered system provides a conceptually new approach to biomass processing—one in which the solvent medium self-assembles from biomass-derived molecules, enabling simplified workup, reduced solvent use, and selective stabilization of reactive intermediates. The present system also helps bridge a gap in current knowledge by offering a molecular-level explanation for previously reported enhancements in HMF stability and yield of HMF extraction from biomass in the presence of levulinic acid, thus expanding the scope of biobased, low-melting liquids beyond traditional DESs.

## Results and Discussion

### HMF-LEV: Solid–Liquid Equilibrium Phase Diagram

In a previous study focused on the solubility and stability of HMF in different kinds of deep eutectic mixtures,^19^ it was noticed that the presence of LEV greatly enhanced the solubility of HMF regardless of the associated hydrogen bond acceptor (HBA). Upon cooling to room temperature, the formulation remained in the liquid state, therefore indicating the formation of stable low-melting mixtures. Therefore, it was decided to thoroughly examine the SLE of the HMF-LEV system and report the complete phase diagram.

The experimentally determined SLE phase diagram of the HMF-LEV binary system over a range of HMF mole fractions (0.12 ≤ *x*_HMF_ ≤ 0.91) is presented in Figure 2a, while the associated DSC thermograms are presented in Figure 2b. Most of the measured mixture compositions exhibit two distinct thermal events: an initial solidus transition, followed by a liquidus peak, demonstrating that the binary HMF-LEV mixture behaves as a simple eutectic system. The solidus temperatures (blue diamonds in Figure 2a) were determined from the onset of the first endothermic peak, and the liquidus temperatures (blue circles in Figure 2a) were determined from the maximum of the second peak in the thermograms (Figure 2b). At *x*_HMF_ = 0.38, a low glass-transition temperature (*T*_g_ = 241.75 K), marked with a blue circle in Figure 2b, is observed. Note that the eutectic peaks used to determine the solidus temperature (blue diamonds in the phase diagram) appear slightly broadened due to the supercooling behavior of levulinic acid, which hinders crystallization. When levulinic acid is cooled below its melting point without crystallizing, it enters a supercooled state. Upon further cooling, when the molecular mobility becomes so limited that crystallization can no longer occur, the system undergoes a glass transition, forming an amorphous solid. As an example, Figure 2c shows the glass transition *T*_g_ = 246.84 K of the sample with *x*_HMF_ = 0.12, which disappeared after extended annealing. In the composition range 0.33 ≤ *x*_HMF_ ≤ 0.63, only a single thermal event is observed in the DSC thermograms, likely due to the close melting temperatures of pure HMF (308.15 K) and LEV (306.15 K), which cause an overlap between the solidus and liquidus transitions. As a result, the exact composition of the eutectic point cannot be determined from DSC thermal analysis alone.

**Figure 2:**
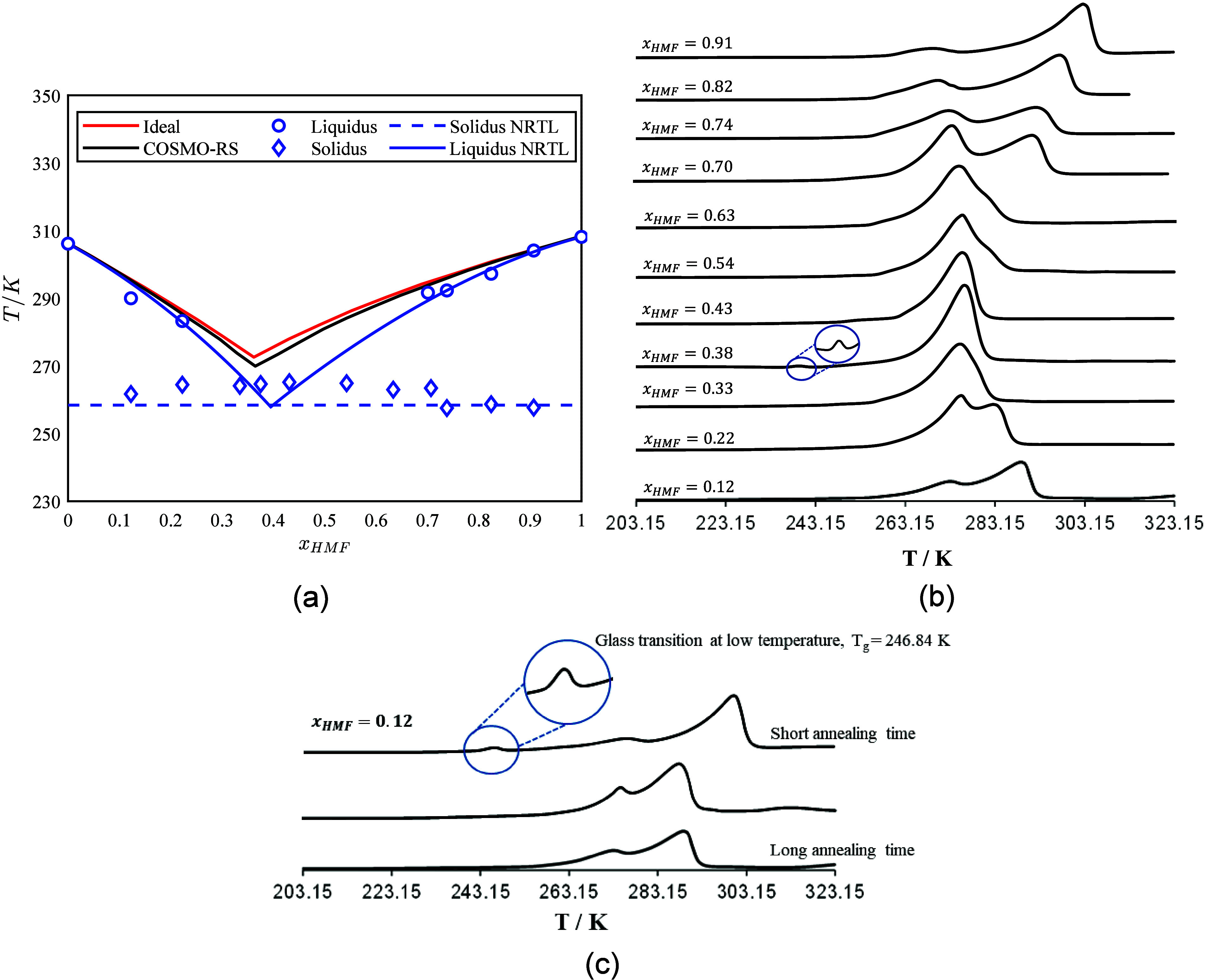
(a) SLE phase diagram of the HMF-LEV system. Blue circles: liquidus temperatures; blue diamonds: solidus (eutectic) temperatures. Solid blue line: NRTL-modeled liquidus; dashed blue line: NRTL-modeled solidus. Solid red line: ideal liquidus. Solid black line: COSMO-RS liquidus. (b) DSC thermograms of the HMF-LEV mixtures at different compositions. (c) DSC curves of the HMF-LEV mixtures at *x*_HMF_ = 0.12 after different annealing times.

To identify the eutectic composition and temperature of the HMF-LEV system, the HMF and LEV liquidus lines were calculated with Equation S3 (see Materials and Methods section-Supporting Information) and using the HMF and LEV melting properties reported in Table S1. The HMF and LEV activity coefficients were calculated with the correlative nonrandom two-liquid (NRTL) activity model (Equations S5–S7).^20^ First, the NRTL model binary interaction parameters were fitted to the experimental HMF and LEV liquidus data points (the blue circles in Figure 2a, Table S2). Then the NRTL model was used to calculate the full SLE phase diagram. The estimated HMF-LEV system eutectic point from the intercept of the calculated HMF and LEV liquids lines (blue solid lines, Figure 2a) is *x*_HMF_ = 0.39 and T = 258 K.

The obtained NRTL model binary interaction parameters and calculated infinite dilution activity coefficients (ln γ_i_^∞^ for *x*_*i*_→0) of components in the binary systems at 298.1 K are reported in Table S3. In a binary system, ln γ_1_^∞^ represents the affinity of component 1 toward component 2, and vice versa ln γ_2_^∞^ represents the affinity of component 2 for component 1.^21^ In HMF-LEV systems, a strong negative deviation from ideality was observed, as indicated by the negative values of ln γ_1_^∞^ and ln γ_2_^∞^. This reflects the favorable HMF-LEV interactions.

To visualize the HMF-LEV system deviation from ideal system behavior, the HMF and LEV liquid lines were calculated with Equation S2 assuming ideal solution (γ_1_ = γ_2_ = 1 – the red solid line in Figure 2a). The experimentally measured liquidus temperatures are lower than those calculated assuming an ideal system, indicating the negative deviation from ideality, which is the key feature of DES.

In addition, to address the question if a predictive thermodynamic model can be used for the determination of the HMF-LEV systems eutectic point, the HMF and LEV solubility lines were calculated with the COnductor-like Screening MOdel for Real Solvents (COSMO-RS) model^22^ (black lines - Figure 2a). The COSMO-RS model, being predictive and based solely on the molecular structure and the corresponding surface charge (σ) profiles, does not fully capture the extensive hydrogen-bond network between HMF and LEV which leads to a strong negative deviation from ideality. In contrast, the NRTL model, although correlative, uses experimental SLE data and is therefore able to accurately account for the observed nonideality of the system.

### Intermolecular Interactions in the HMF-LEV Systems

The liquid mixtures here considered for the ^1^H NMR spectroscopic investigation were obtained for an array of compositions spanning the 0.05 ≤ *x*_HMF_ ≤ 0.95 range and all having water contents in the range 0.3–1 wt % (Table S4), which can be reasonably assumed to mimic a close-to-real condition. A first indication of the relative change in the strength of the intermolecular forces was assessed through the analysis of chemical shifts’ variation of the individual protons, defined as Δδ (ppm) = δ_mix_ – δ_pure_, as a function of the mixture composition. We have already successfully used such an approach for the assessment of liquid structuration in eutectic systems.^23^ The results are summarized in Figure 3a,b.

**Figure 3:**
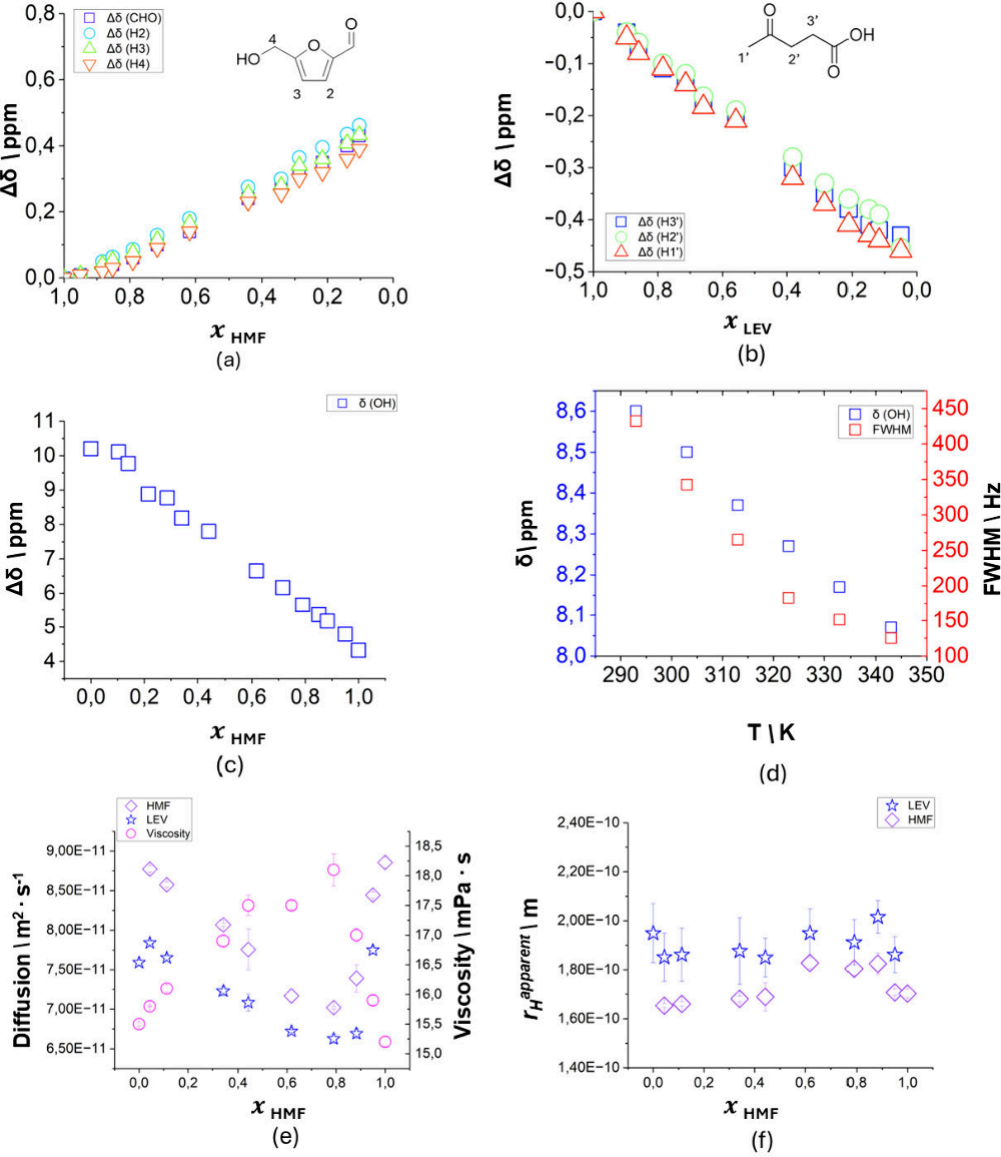
(a) ^1^H NMR chemical shift variation at 313 K of HMF signals with respect to HMF molar fraction in HMF-LEV mixtures. (b) ^1^H NMR chemical shift variation at 313 K of LEV signals with respect to LEV molar fraction in HMF-LEV mixtures. (c) Chemical shift (δ - ppm) of the −OH signal with respect to *x*_HMF_. (d) Chemical shift (δ - ppm) of the medium −OH signal and full width at half-maximum (FWHM – Hz) with respect to temperature of the eutectic mixture. (e) Diffusion coefficients and viscosity measured at 313 K for the binary mixtures at different molar fractions. (f) Apparent hydrodynamic radius over the compositional range, error bars were calculated using the standard error-propagation theory.

For all the nonexchangeable HMF protons, a chemical shift increase is observed upon LEV addition (Δδ > 0, or deshielding effect). As demonstrated in a previous study on progressive dilution of HMF with either a noninteracting (CD_3_CN) or interacting (DMSO) solvent,^24^ this nonselective downfield shift of the aromatic and methylene protons is consistent with the progressive disruption of the π-π stacking characteristic of pure liquid HMF, which reduces the paramagnetic effect of aromatic electrons. A downfield shift is also observed for the aldehyde proton, where the establishment of new HMF-LEV interactions partially compensates the close paramagnetic effect of aromatic electrons, resulting is a slightly less intense shift. Concurrently, all the nonexchangeable proton signals of LEV experience Δδ < 0 upon HMF addition (Figure 3b), indicative of increased shielding, likely arising from the progressive weakening of LEV-LEV H-bonds and replacement with HMF-LEV interactions, introducing anisotropic π-currents effects. Notably, the signals corresponding to the exchangeable protons of LEV (−COOH) and HMF (−OH), appear at 10.2 and 4.32 ppm in the spectra of the pure components, respectively, and merge into an average signal in all binary mixtures. This is very common in both deep and (close-to-) ideal eutectic mixtures, and indicates fast exchange in the NMR time scale.^25^ Overall, the Δδ­(OH) values of the exchangeable protons (red diamonds, Figure 3a) undergo deshielding upon dilution of pure HMF with increasing amount of LEV. This is consistent with a progressive replacement of the weak HMF-HMF H-bonds with the stronger HMF-LEV and LEV-LEV ones. Both chemical shift and full-width-at-half-maximum of average OH/COOH signal of the eutectic composition were monitored upon heating from 298 to 343 K (Figure S12 and 3d). A progressive upfield shift and line narrowing is observed, clearly indicating a weakening of the H-bond involving the corresponding protons with temperature and an increase of the exchange rate. Following a previous study,^25^ the relative difference between the chemical shift of the exchangeable proton at 60 and 25 °C (ΔδOH = |δOH­(60 °C) – δOH­(25 °C)|) can be used as a descriptor of the H-bond strength. ΔδOH = 0.41 ppm is observed here for HMF-LEV 1:2, which overall positions the new eutectic mixture among the systems with a strong and robust H-bond network.

Further details of the solvation features were obtained by investigating the diffusivity of the single components as a function of the system composition. The self-diffusion coefficients of the mixture components were measured by PFG NMR experiments and are reported in Figure 3c for the selected compositions, together with the corresponding viscosity data. All data were acquired at 313 K, to allow a comparison with the pure components in liquid state. It is evident that the self-diffusion coefficients of the two pure components are greater than those of their mixture. The diffusion coefficients of both HMF and LEV decrease as the components are mixed, reaching a minimum at *x*_HMF_ = 0.8. In a symmetric way, the viscosity exhibits the opposite trend, with lower viscosity values corresponding to the pure substances. Upon mixing, the viscosity increases, reaching a maximum at *x*_HMF_ = 0.8. The negative infinite dilution activity coefficients (ln γ_1_^∞^ = – 1.0744 and ln γ_2_^∞^ = – 1.4366, see Table S3) indicate strong mutual affinity between HMF and levulinic acid. While these values are not extremely negative, they are sufficiently below zero to reflect moderately strong, favorable interactions between the components. These interactions are consistent with the observed decrease in self-diffusion coefficients and increase in viscosity upon mixing (Figure 3c). However, the persistence of separate diffusion coefficients for the HMF and LEV suggests that these interactions are transient and not strong enough to result in stable supramolecular aggregates. The analysis of experimental density data reveals negative excess molar volumes (*V* ^E^), indicating a significant volume contraction driven by strong specific interactions, particularly hydrogen bonding, between HMF and LEV. This strong molecular attraction is further confirmed by positive viscosity deviations (Δη), which suggest the formation of a rigid network that resists flow more effectively than the pure components. The data provide an important methodological indication in discussing ideality vs nonideality in eutectic mixtures, calling for multidisciplinary cross-check.

The apparent hydrodynamic radius (*r*_*H*_^*apparent*^) can be computed from the Stokes–Einstein equation combining diffusivity and viscosity data (Figure 3d). A slight, yet non-negligible, increase in *r*_*H*_^*apparent*^ of HMF with respect to its pure form can be observed for mixtures with low LEV content. This is compatible with a scenario where the intermolecular interactions between the sea of HMF molecules and the scarcer LEV species overall stabilize the network, restricting their motion, and increasing their apparent size. Compositions in the range 0.6 < *x*_HMF_ < 0.9 show an increase of *r*_*H*_^*apparent*^ of both HMF and LEV. In this composition range it is reasonable hypothesize that π-π stacking of HMF and HMF-LEV hydrogen bonds are contributing to the HMF and LEV solvation shells, also in agreement with the chemical shift titrations discussed previously, with unselective increase of the hydrodynamic radii of both components.

### Modeling of the HMF-LEV Liquid Mixtures

The network of intermolecular interactions was initially investigated via DFT calculations^26^ considering the following minimal clusters: 1HMF:1LEV, 1HMF:2LEV, and 2HMF:1LEV, and compared with the ones of the pure precursors (1HMF:1HMF and 1LEV:1LEV minimal clusters). DFT calculations revealed the formation of hydrogen bonded clusters with remarkably large interaction energies (Δ*E,* Table S5). Based on the Δ*E* values, the strongest interacting molecular clusters are those corresponding to the eutectic composition (1HMF:2LEV). HMF and LEV self-aggregation revealed well-interconnected pairs with large Δ*E* values (Figure S3). QTAIM,^27^ NCI^28^ and COSMO-RS analyses confirmed the role of hydrogen bonds and significant van der Waals interactions (Figures S4 and S5) largely localized in the inter-ring region for the HMF:HMF cluster, in agreement with our data on pure HMF.^23^ The nonpolar interactions progressively lose importance in the 2HMF:1LEV cluster, thus confirming that structural model postulated on the basis of the NMR data, i.e., the downfield shift of the exchangeable hydroxyl NMR signal, attributed to HMF-LEV hydrogen bond. These findings support the primary role of H-bonds in the HMF-LEV interaction, leaving a minor role to the nonpolar interactions.

As mentioned in the Introduction, part of the present investigation is also related to uncovering the interaction of water molecules with the HMF-LEV system. Indeed, the experimental characterization described in the first section considered low-hydration HMF-LEV mixtures, with the aim of mimicking more closely real operating conditions on nonanhydrous HMF-LEV biomass feeds. This prompts *in-silico* simulations of the HMF-LEV-H_2_O mixtures. In general, the effect of water on the HMF-LEV clusters’ hydrogen bonds is negligible (Table S5), with two exceptions: HMF­(H1)-LEV2­(O6) and LEV­(H5)-HMF1­(O2) H-bonds were disrupted in the 1HMF:2LEV and weakened in the 2HMF:1LEV systems (Figure 1a for atom labeling) due to the competitive insertion of water in the static cluster, as water competes for the donor site. Despite the extinction of these H-bonds, QTAIM analysis reveals a compensatory strengthening of the remaining interactions (increased ρ and ∇^2^ρ), reinforcing the overall 1HMF:2LEV and 2HMF:1LEV network, respectively. These results disclose the robustness of H-bond network in the 1HMF:1LEV and 2HMF:1LEV aggregates, contrasting 1HMF:2LEV H-bond lessening, upon water addition. The stability of HMF:LEV+water systems was first assessed in terms of interaction energy, *E*_int(HMF:LEV‑water)_, and these values become more negative (i.e., higher stability) with the addition of water molecules. The formation of water–water H-bonds, and water clusters around the HMF-LEV aggregates, undoubtedly indicates that the water molecules prefer to interact with each other rather than surround HMF-LEV cluster. Second, the effect of adding water molecules was evaluated by calculating the interaction energy contribution per water molecule, defined as *E*_int(HMF:LEV‑water)_/*n*_water_ (Figure S6), to determine whether the effect is additive, synergistic, or antagonistic. For all the clusters, the addition of up to 2 water molecules exhibits a synergistic effect on the interaction energy. However, the addition of a third, and subsequent, water molecule leads to an antagonistic effect. 1HMF:2LEV system is an exception, with lower *E*_int(HMF:LEV‑water)_/*n*_water_ values (more energetically stable, Figures S3 and S6 and Table S5).

The nanoscopic interactions of the HMF:LEV mixture, as well as the water effect from moisture absorption on the liquid mixtures, were evaluated using MD simulations (see Tables S9 and S10 for system configuration details and water concentration).^28^ Radial distribution functions (*rdf*) were computed for all atomic pairs and systematically analyzed using the connection matrix (*cmat*) function as shown in Figure 4 and Figure S7.

**Figure 4:**
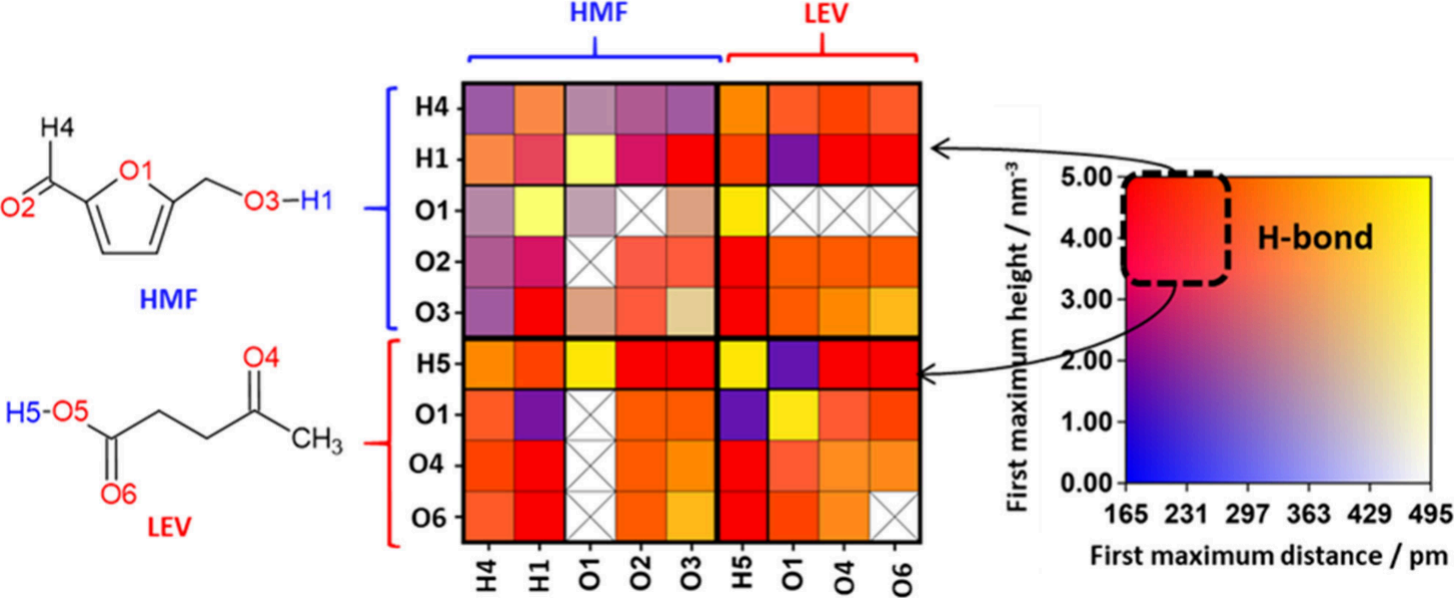
Connection matrix, *cmat*, for the 1HMF:2LEV system at 313 K. In each square, the intensity and distance of the first maximum of the corresponding RDF is represented by the colormap. This is a simplified example of a *cmat* considering only the atoms involved in the hydrogen bonds. Please refer to the Supporting Information for the complete analysis.

*Cmat* results highlight three key features: (i) HMF-LEV interactions primarily occur through HMF­(O2) and HMF­(O3) acceptor sites with LEV­(H5) donor site, and LEV­(O4) and LEV­(O6) acceptor sites interacting with HMF­(H1), in line with DFT calculated hydrogen bond strengths (Figure S4); (ii) HMF and LEV molecules self-associate via HMF­(O2)-HMF­(H1), and LEV­(O4)-LEV­(H5) and LEV­(O6)-LEV­(H5); (iii) all donor sites compete with water (Hw) in the hydrated systems. The HMF-LEV radial distribution functions (*rdf* or *g*(*r*), Figure 5) reveal pronounced and sharp peaks for HMF­(O3)-LEV­(H5) and LEV­(O4)-HMF­(H1) interactions, with donor–acceptor distances consistent with DFT-derived values (Figure S4). Notably, these specific interactions yield the highest coordination numbers, *CN*, confirming the formation of a stable and well-interconnected HMF–LEV solvation shell (Figure 5).

**Figure 5:**
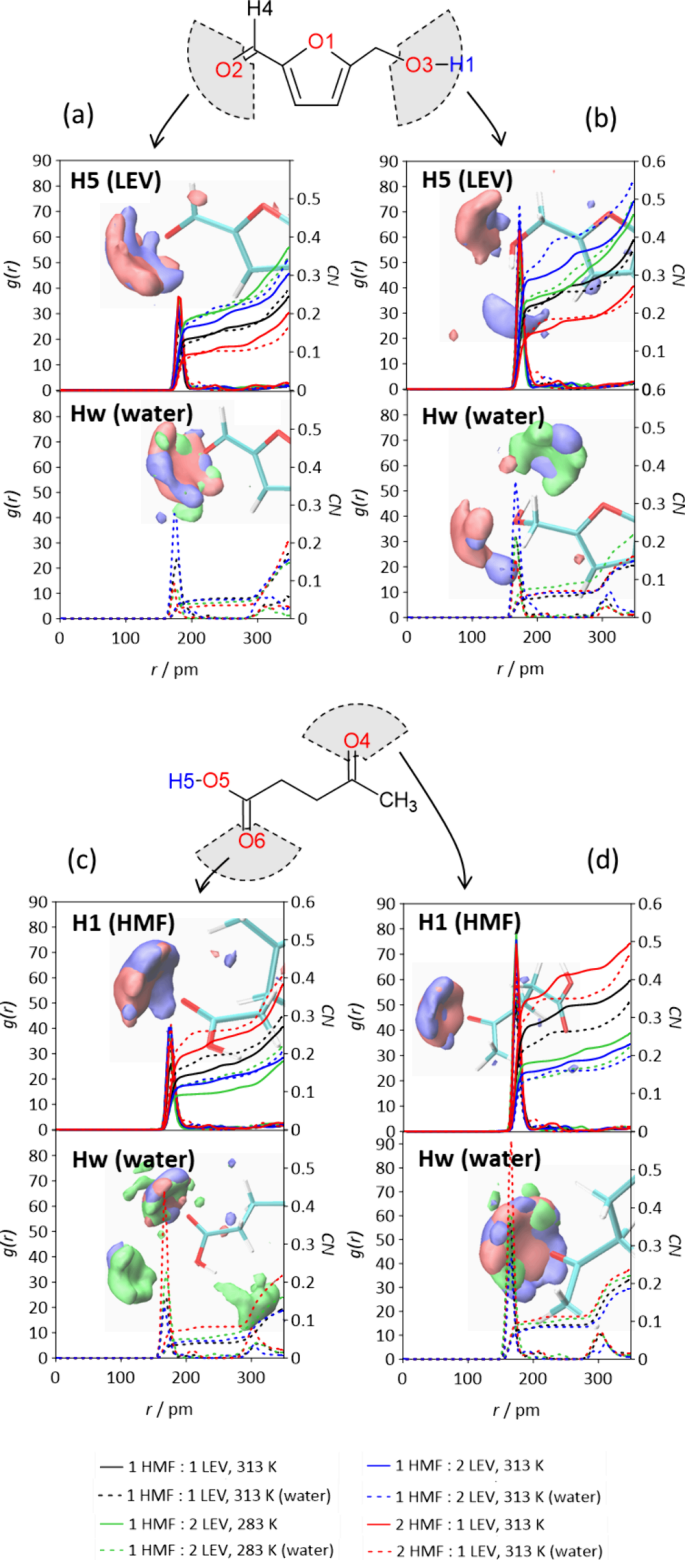
Site–site *rdf, g*(*r*), and coordination numbers, CN, for (a) HMF­(O2), (b) HMF­(O3), (c) LEV­(O6) and (d) LEV­(O4) acceptor sites and hydrogen LEV­(H5), HMF­(H1) and water (Hw) donors. Atom labeling as in Figure[Fig fig1]b. Figures show *sdf* of 1HMF:2LEV systems at 313 K. Isosurface color code: blue for HMF, red for LEV and green for water. Isosurface value: 0.25.

These interactions do not weaken upon water addition, thus confirming the resilience of the HMF-LEV liquid mixture to the presence of water and paving the way to actual applications of the HMF-LEV systems in biomass treatment. HMF and LEV interactions with water are confirmed by the *g­(r)* of HMF­(O2), HMF­(O3), LEV­(O4) and LEV­(O6) shown (Figure 5a–d, bottom panels). Spatial distribution functions (*sdf*) indicate, for all the systems, HMF-LEV hydrogen bonds dominating over HMF and LEV self-interaction (red and blue spots, Figure 5, Table S6). Moreover, *sdf* and *N*_H‑bonds_ reveal the competing effect of water molecules with LEV and HMF donor sites (green spots - Figure 5). Water effect on HMF-LEV intermolecular interactions shows negligible impact on *N*_H‑bonds_ values, for all the HMF:LEV molar ratios and temperature (Table S6). Noteworthy, HMF-LEV *N*_H‑bonds_ values become higher for the eutectic composition with water, in contrast with ab initio calculations outcomes. This might be due to the establishment of water-HMF and water-LEV H-bonds (confirmed by *rdf* and *sdf*, Figure S8) that weaken HMF-HMF and LEV-LEV H-bonds, thus releasing new available acceptor and donor sites for the setting of HMF-LEV H-bonds. In fact, the number of HMF­(O2)-HMF­(H1) and LEV­(O6)-LEV­(H5) bonds decrease upon water addition, while LEV­(O6)-HMF­(H1), HMF-water and LEV-water number H-bonds increase. This effect is remarkably larger at the eutectic composition. To verify the HMF-HMF structural disruption, the combined distribution functions, *cdf*, correlating HMF center-of-ring (CoR) distaces and angle orientation between HMF ring planes were analyzed (Figure S9). In high HMF concentrated systems, high-probability regions corresponding to parallel π–π stacking (angles of 0° or 180° and distances of ≈3.8 Å) are localized, while for increasing amounts of LEV, the probability of these stacking interactions diminished notably. This confirms the disruptive effect of the LEV.

This conclusion is supported by the analysis of the intermolecular interaction energies (*E*_int_) for the HMF:LEV systems with and without water molecules, which indicates that HMF-LEV interaction predominates over all the others possible combinations (Figure 6a). Noticeably, *E*_int_ of HMF-LEV at eutectic composition is significantly high (−118.3 and −121.64 kJ mol^–1^ at 283 and 313 K), with further improvement in the presence of water (−130.5 kJ mol^–1^ at 313 K). Low water–water *E*_int_ values lead to highly dispersed water molecules distribution along the fluid, opposite to what was observed via DFT. Water molecules lean toward HMF-water and LEV-water interaction, consistent with previous formulation. Furthermore, interaction energy values *E*_int_(HMF-water) > *E*_int_(HMF-HMF) and *E*_int_(LEV-water) ≈ *E*_int_(LEV-LEV) draw the conclusion of HMF-LEV hydrogen bond network enhanced in the presence of water due to LEV-LEV and HMF-HMF self-association distortion. The difference between DFT and MD results comes from the fundamental limitations of each method.

**Figure 6:**
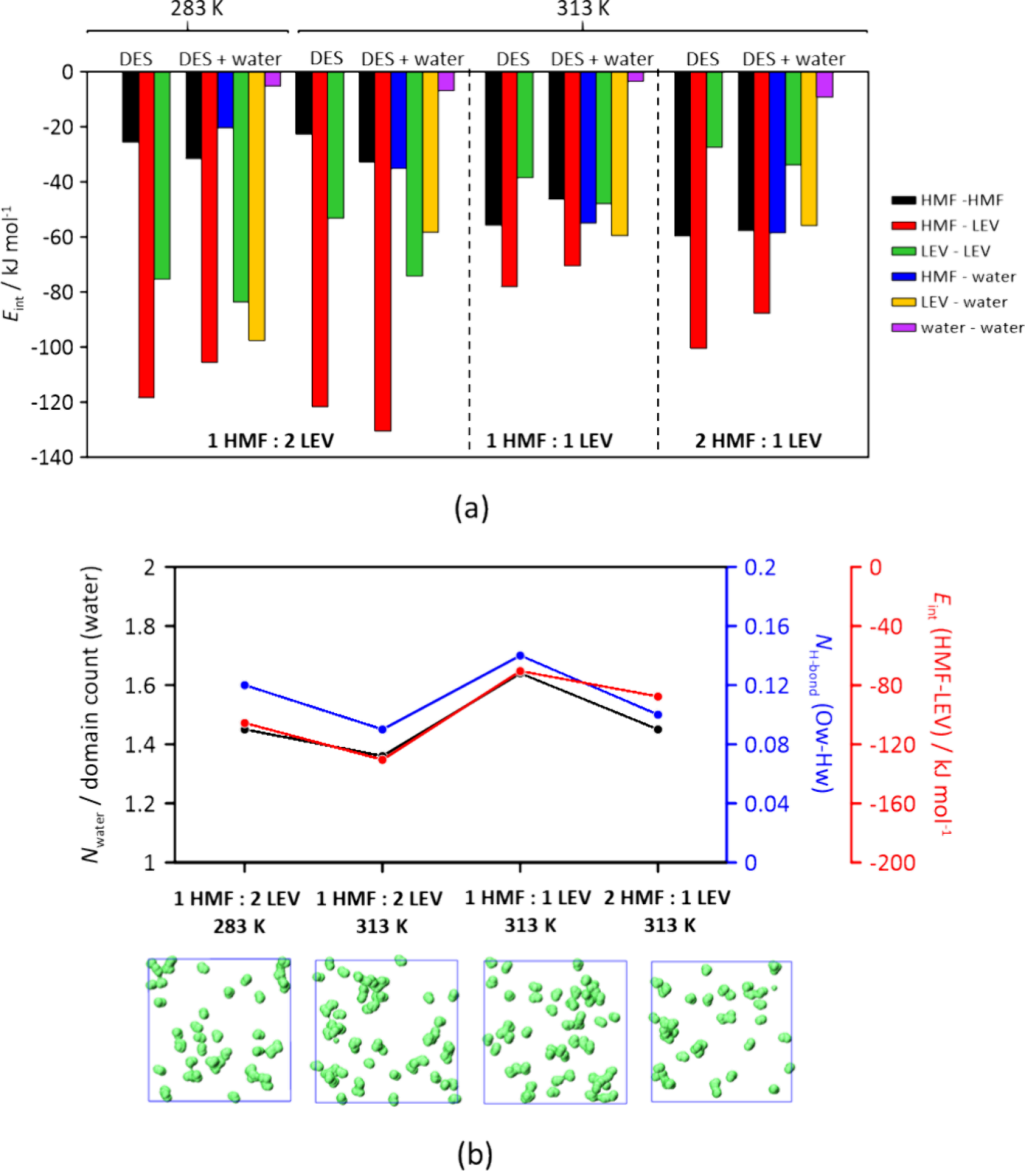
(a) *E*_int_ of all possible intermolecular interactions from MD simulations at 283 and 313 K for the HMF:LEV considered systems; (b) Water molecules per domain number, *N*_water_/domain count (water), black; number of Ow-Hw hydrogen bonds, *N*_H‑bonds_ (Ow-Hw), blue; and HMF-LEV *E*_int_, red, of HMF:LEV-water mixtures from MD. Figures show water molecules (green spots) distribution within the simulation boxes in the last simulation frame.

MD simulation can handle a large system that realistically represents the bulk liquid with a true water concentration; however, DFT calculation is limited to a very small number of molecules. The number of water molecules in the DFT clusters are not meant to represent real concentration but a computational model to approximate how water molecules, in the immediate vicinity, might interact with the HMF and LEV molecules.

Finally, the domain analysis of molecular species gave an indication of the system microheterogeneity. While HMF and LEV molecules represent one connected domain (count = 1), water domain analysis (domain counts >25, Table S7) shows nonconnected, spatially dispersed water molecules, discarding water clusterization within the liquid. Low water–water interaction indicators (*N*_water_/domain count and water–water *N*_Hbond_, black and blue lines; Figure 6b) are correlated with high HMF-LEV interaction index (HMF-LEV *E*_int_, red in Figure 6a). Therefore, this would confirm the reinforced effect of water over the HMF-LEV interaction and its fluctuation according to the HMF:LEV molar ratio, revealing the implication of water–water interactions on a 1:2 eutectic mixture. These results are supported by dynamic properties (diffusion coefficients, Table S8; velocity distribution functions, Figure S10), revealing higher HMF and LEV mobility at the eutectic composition. High water mobility (*D*_*H*_2_ *O*_ ≫ *D*_*HMF*_ > *D*_*LEV*_) in LEV-rich mixtures confirms a dynamic lubricating role, water acting as spacer that disrupts HMF-HMF and LEV-LEV associations. On the contrary, the diffusion coefficient of water drops to a lower value in the HMF-rich mixture. This suggests a specific interaction where water may get trapped within the HMF-HMF network. Overall, the structural analysis revealed HMF-LEV mixtures with robust, well-interconnected hydrogen bond network with negligible water effects on the intermolecular structure and with particular attention to eutectic system, where the presence of water molecules showed to be correlated with the strengthening of HMF-LEV interactions.

Finally, we note that the geometric parameters derived from DFT (Table S5) and the H-bond statistics obtained from MD (Table S6) are not directly comparable due to the different physical regimes modeled. The DFT results describe the intrinsic electronic properties of static, energy-optimized clusters in the gas phase (0 K), dominated purely by enthalpic interactions. In contrast, MD results reflect the macroscopic bulk solvent at a finite temperature, where entropic contributions and thermal fluctuations govern the system. Consequently, a distorted H-bond evidenced in static DFT may still exhibit high probability of occurrence in the dynamic liquid phase (MD simulations) due to the stabilizing solvation effects

### System Stability

To validate the feasibility of extracting 5-hydroxymethylfurfural (HMF) from lignocellulosic biomass using levulinic acid (LEV), we first assessed the intrinsic stability of the HMF-LEV deep eutectic system. The binary mixture (1:2 molar ratio – eutectic composition) was monitored over time (up to 30 days) and upon thermal treatment at 60 °C for 48 h, as reported in Figure 7.

**Figure 7:**
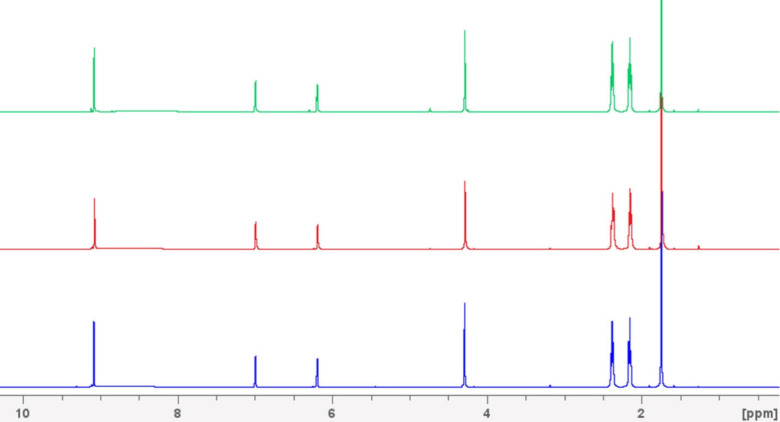
^1^H NMR spectra of the HMF/LEV (1:2) mixture. Bottom trace (blue): freshly prepared (t_0_). Middle trace (red): after 30 days (t_30_). Top trace (green): after 48 h at 60 °C.

No additional resonances or signal broadening were observed in the ^1^H NMR spectra, confirming that the HMF-LEV system remains chemically stable under the tested conditions. In particular, no new peaks associated with HMF degradation (e.g., formation of humins or ring-opened species) nor esterification between the hydroxyl group of HMF and the carboxylic function of levulinic acid were detected, even upon heating. This stability is essential for enabling LEV to act simultaneously as a solvent and extraction medium without inducing side reactions that compromise the HMF integrity. On the other hand, HMF exhibited lower stability in commonly employed green solvents such as ethyl acetate and methyl ethyl imidazolium acetate (used here as a representative ionic liquid). In both cases, the chemical stability of 5-hydroxymethylfurfural was limited, as evidenced by the formation of a precipitate within a few hours in ethyl acetate and within a few days in the ionic liquid (see Figures S13 and S14).

The thermal stability of the pure components and the DES herein reported was investigated by means of TGA measurements under both nitrogen and air flux. The resulting TGA and DTGA curves are reported in Figure S15, while the values of temperatures at 3% (T_3%_) 5% (T_5%_) and 10% (T_10%_) mass loss, the residue at 500 °C (R_500 °C_) and the maximum mass loss derivative temperature (T_DTGA(max)_) are reported in Table S12. Overall, the results obtained showed that no significant weight loss was observed for the HMF-LEV eutectic system up to ∼132 °C (air) and ∼144 °C (N_2_), resulting in an increased stability with respect to the pure components.

### HMF-LEV Liquid Phase for HMF Extraction from Biomass: Proof of Concept

To provide a proof of concept that the formation of a liquid HMF-LEV phase can enable a solventless strategy for biomass processing, we performed a controlled extraction experiment. Instead of working with real lignocellulosic residues after dehydration—a scenario that lies beyond the scope of this study—we used a synthetic biomass surrogate based on the composition reported by Krasznai *et* al.^29^ A mixture containing 45 wt % cellulose, 30 wt % hemicellulose, and 25 wt % lignin was prepared from commercially available powders.

To this solid matrix, 200 mg of HMF were added, and the mixture was manually ground in a mortar until complete homogenization was achieved. Different solvents were then tested to compare the extraction efficiency (EE, i.e., (mol_HMF‑extracted_/mol_HMF‑real_) × 100) of solid, anhydrous levulinic acid with that of traditional solvents (Figure 8). Upon addition of LEV, simple manual mixing at room temperature rapidly produced a dark brown liquid phase. This observation confirms the spontaneous formation of a liquid HMF-LEV phase in the LEV-rich environment, even without controlling the stoichiometric ratio. This liquid phase could be separated from the solid residue by centrifugation, without resorting to conventional solvent extraction or dissolution in water.

**Figure 8:**
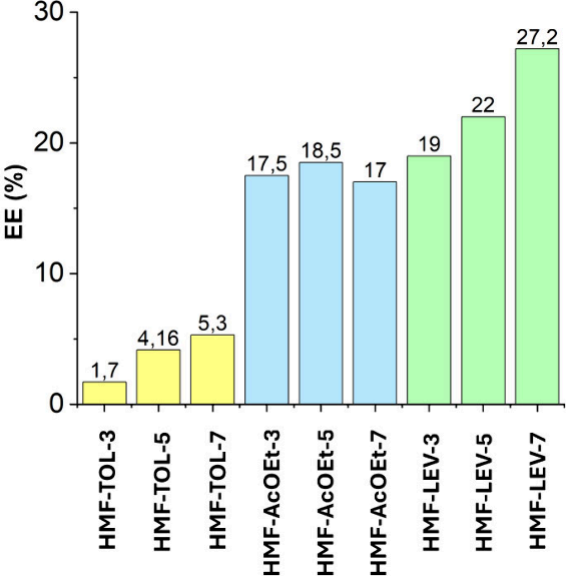
Extraction efficiency (EE) of HMF from biomass proxy with respect to different solvents. Biomass proxy fixed on 1 g scale; HMF quantity fixed to 200 mg. Sample codes are defined as HMF-Solvent-X, where X indicates the amount (g) of solvent used in a single extraction.

The extraction efficiency (EE) was determined for single-stage solid–liquid extractions performed using 3, 5, and 7 g of each solvent. In the case of toluene (yellow bars in Figure 8), the EE ranged from 1–5%, consistent with the very low solubility of HMF in nonpolar aromatic solvents and therefore indicative of poor extraction performance. Ethyl acetate provided an average EE of approximately 18%, with only minor variation as a function of solvent mass (blue bars). When levulinic acid was employed as the extracting phase, the EE increased to 19–27%, demonstrating a significantly stronger affinity of LEV for HMF compared to the other two commonly used extraction solvents (green bars). This enhanced performance after a single extraction step is plausibly attributed to the *in situ* formation of an HMF-LEV deep eutectic system (DES), whose stronger intermolecular interactions could promote increased HMF solubility in the levulinic acid phase, resulting in fact in the formation of a liquid phase from the union of the two solids. In this system, a direct correlation between the amount of LEV used and the resulting EE was observed, further supporting the favorable interaction between the HMF and the LEV.

Finally, it is worth providing an initial evaluation of the greenness of potential applications of the HMF-LEV system. To this end, we adopted an approach inspired by Gałuszka’s Analytical EcoScale,^30^ defining a set of descriptors associated with penalty points. The procedure is summarized in Table 1, where some descriptors are reported for levulinic acid, toluene, and ethyl acetate, considering their use as solvents/extractants in the final step of HMF obtainment from biomass. An additional descriptor —the vapor pressure at the given temperature, was also introduced as an indicator of the solvent’s volatility and VOC-related impact.

**Table 1:** Summary of Descriptors for Analytical EcoScale Evaluation of Three Solvents/Extractants

Solvent	Number of pictograms	Signal word	Penalty points (PPs)	H-phrase	Vapor pressure (atm) (T (K))
Toluene	3	danger	6	H225 H304 H315 H336 H361d H373 H412	3.737 × 10^–2^ (298)^31^
Ethyl Acetate	2	danger	4	H225 H319 H336	1.226 × 10^–1^ (298)^32^
LEV	2	danger	4	H225 H319 H336	1.153 × 10^–2^ (328)^33^

The data in Table 1 show that ethyl acetate and levulinic acid have comparable Analytical EcoScale penalty points in terms of reagent toxicity, as well as the same hazard statements. However, the markedly lower volatility of levulinic acid represents a significant advantage, particularly when considering the potential implementation of an HMF-LEV liquid phase in future biomass processing (see the Conclusion).

## Conclusion

The HMF-LEV system forms a stable liquid at room-temperature across a wide compositional range, with a eutectic point near *x*_HMF_ ≈ 0.39. Solid–liquid equilibria calculated using the correlative NRTL activity model reveal strong negative deviations from ideality, a hallmark of deep eutectic systems. NMR spectroscopy, viscosity measurements, and thermal analyses confirm the establishment of intense intermolecular interactions at the eutectic composition. LEV plays a central role in this structuration through its dual function as hydrogen bond donor and acceptor, enabling efficient bridging with HMF functional groups.

DFT and molecular dynamics simulations identify dominant HMF-LEV hydrogen bonds that override the self-association effects. The combined experimental and theoretical evidence demonstrates that the HMF-LEV binary system fulfils the established physicochemical criteria for deep eutectic solvents:^34^ (i) a eutectic melting temperature significantly lower than predicted for an ideal mixture, (ii) strong intermolecular hydrogen bonding between a hydrogen bond donor and acceptor, and (iii) pronounced nonideality in the liquid phase. The mixture remains liquid over a large composition range and retains structural integrity in the presence of small amounts of water.

Notably, at the eutectic composition, water enhances the hydrogen-bonding framework by selectively weakening LEV-LEV and HMF–HMF associations, thereby promoting cross-species bonding. With strong hydrogen bonding capacity, thermal stability, and water resilience, this bioderived deep eutectic system offers a promising platform for the development of green solvent technologies and sustainable biomass valorization strategies.

Overall, this work demonstrates that the newly discovered HMF-LEV eutectic mixture showed excellent chemical stability under both long-term storage and thermal stress, ensuring that HMF integrity is preserved. Preliminary extraction tests highlighted the ability of LEV to form an *in situ* liquid DES phase with HMF allowing for substantially higher extraction efficiencies than conventional solvents, even in a heterogeneous biomass surrogate, thereby reducing dependence on external organic solvents. These findings highlight that levulinic acid—typically generated as a byproduct during HMF formation, can be repurposed as an effective medium for HMF extraction, opening the door to future studies aimed at integrating this valorization strategy into more comprehensive and resource-efficient biorefinery schemes. Notably, an HMF-LEV mixture has already been used directly, without prior separation, in a one-pot catalytic oxidation/hydrogenation system converting HMF to 2,5-furandicarboxylic acid (FDCA) and levulinic acid to γ-valerolactone (GVL).^35^ The fact that catalytic upgrading can proceed in such mixtures, together with our observation of spontaneous liquid-phase formation, further supports the view that HMF-LEV systems represent realistic and promising platforms for future biorefinery applications.

## Experimental Section

### Materials and Methods

5-Hydroxymethylfurfural (HMF, ≥99%) and levulinic acid (≥99%) were purchased from Sigma-Aldrich (Sigma-Aldrich, St. Louis, USA). HPLC-grade water was purchased from VWR Chemicals (VWR International, Radnor, USA). All chemicals were used as received without further purification. The water content was measured in triplicate using the Karl Fisher titration method performed on an MKC-710 B instrument by KEM Kyoto Electronics. Viscosity was measured in triplicate at 40 °C, using an Anton Paar MCR502 rheometer with a cone–plate configuration (50 mm diameter, 1° angle, and 99 μm truncation).

The density (ρ) of HMF:LEV mixtures was determined at different temperatures and molar ratios using a vibrating-tube densimeter (Anton Paar DMA 1001, uncertainty ± 1 × 10^–4^ g cm^–3^).

TGA analyses were performed with a Mettler Toledo TGA2 instrument. Samples’ weight ranged from 5 to 20 mg; as sample holders were used alumina crucibles. The analyses were carried out with a program that provides a single heating cycle from 30 to 500 °C at 20 °C/min under nitrogen or air atmosphere (50 mL/min).

NMR measurements were performed at 313 K without sample spinning with a Bruker NEO 500 console (11.74 T, ^1^H resonance frequency of 500.13 MHz) equipped with a direct observe BBFO (broadband including fluorine) iProbe and a variable-temperature unit. The pure mixtures were transferred to a 5 mm NMR tube equipped with a coaxial insert containing deuterated dimethyl sulfoxide (DMSO-d6). The instrument was carefully tuned and shimmed, and the 90° pulses were calibrated. ^1^H self-diffusion coefficients were measured by pulsed field gradient (PFG) NMR experiments by applying sine-shaped pulsed magnetic field gradients along the *z*-direction up to a maximum strength of G = 48.15 G cm^–1^. The diffusion experiments were performed using a bipolar pulse longitudinal eddy current delay (BPP-LED) pulse sequence.

Further details on sample preparation, instrument calibration, data collection, and processing are reported in the Supporting Information.

### General Procedure for HMF-LEV Mixture Preparation

Levulinic acid was placed in a dryer under vacuum for 8 h at room temperature and kept under vacuum overnight. The desired amount of 5-hydroxymethylfurfural and levulinic acid (Table S4) were then weighed in a vial which was then sealed and heated at 35 °C under stirring until a homogeneous orange liquid phase was obtained.

### HMF Extraction from Biomass Proxy

450 mg portion of cellulose (Avicel PH-101), 300 mg of hemicellulose (Xylan), and 250 mg of lignin were weighed, transferred to a mortar, and manually ground to obtain a homogeneous powder of all components. To this, 200 mg of HMF were added and then manually ground again to incorporate HMF into the biomass proxy. The extracting agent, namely, LEV, AcOEt or toluene (TOL), was then added to the mortar and mixed manually. The final suspension was transferred into a falcon and subjected to centrifugation for 30 min. The liquid phase was then separated from the solid one and analyzed via ^1^H-NMR in the presence of 1,4-dimethoxybenzene as a standard to quantify the amount of HMF extracted by a single extraction.

### Computational Methods

The thermodynamic modeling of SLE is reported in detail in the Supporting Information and in equations S3–S9. A conformational search for HMF and LEV molecules was carried out employing COSMOconf (COSMOtherm package, version 24.1.0) and DFT BP/TZVP calculations. Structures, relative energies, and conformer probability distributions are shown in Figure S11. DFT calculations were performed using Orca software,^36^ with B3LYP functional,^37,38^ 6–311++G­(d,p) basis set and D3 semiempirical method.^39^ 1HMF:2LEV, 1HMF:1LEV, 2HMF:1LEV, 1HMF:1HMF and 1LEV:1LEV minimal clusters were constructed according to ABCluster^40^ global optimization using xTB semiempirical method.^41^ Among the six distinct minimal clusters initially evaluated for each HMF:LEV combination, the most thermodynamically stable configurations were selected for further analysis considering the addition of up to 5 H_2_O molecules. The HMF:LEV minimal cluster interaction energies (Δ*E*) were determined as the difference between the total energy of the cluster and the sum of the individual monomer energies. The HMF:LEV-water interaction energies (*E*_int(HMF:LEV‑water)_) were calculated as the difference between the total energy of the cluster and the sum of the HMF:LEV interaction energy and the water monomer energy. To mitigate the Basis Set Superposition Error, the counterpoise correction to the energy was applied.^42^ The characterization of the hydrogen bonding topology was conducted within the Quantum Theory of Atoms in Molecules (QTAIM) framework. Key intermolecular interactions were examined via Bond Critical Points (BCPs), based on electron density (ρ_e_) and the Laplacian of electron density (∇^2^ρ_e_). Additionally, the optimized clusters were subjected to Non-Covalent Interaction (NCI) analysis.^43^

Phase equilibrium properties of the investigated HMF:LEV deep eutectic solvent were predicted using the COnductor-like Screening MOdel for Real Solvents (COSMO-RS) model,^44^ using COSMOtherm. COSMO files for the individual species were generated from the optimized molecular structures with DFT (BP86/def-TZVP) calculations. The provided melting temperatures and fusion enthalpies of pure compounds were 308.20 K and 19.8 kJ mol^–1^ for HMF^45^ and 306.20 K and 9.22 kJ mol^–1^ for LEV.^45^ Four HMF conformers and two LEV conformers were employed according to probability distribution values.

Molecular Dynamics (MD) simulations were carried out using MDynaMix v.5.3 software^46^ and Merck Molecular Force Field,^47^ as obtained from the SwissParam database.^48^ Force field parameters with atomic charges assigned based on ChelpG DFT calculations^49^ are summarized in Table S11. Inferred MD densities were compared with the experimentally measured (deviations of 0.75 to 2.91%), confirming the reliability of MD simulations (Table S9). Further details of the computational procedures are reported in the Supporting Information.

## Supplementary Material



## Notes

Experimental protocols for sample preparation and characterization, including instrumental setup details, the basic theory and equations for thermodynamic modeling, and NMR data processing, details of DFT calculations, COSMO-RS outputs, MD data, lists of thermodynamic data, viscosity, calculated molecular properties, and diffusion coefficients (PDF)
