# Supplementary material for: Stable and Water-Tolerant Deep Eutectic Solvent from Biomass-Derived 5‑Hydroxymethylfurfural (HMF) and Levulinic Acid
Source: ACS Sustain Chem Eng. 2026 Feb 12;14(7):3699–710. doi: 10.1021/acssuschemeng.5c13551 (PMC12934199; doi:10.1021/acssuschemeng.5c13551)
Supplement: Supplementary file 1 [file sc5c13551_si_001.pdf]

# Supplementary Information

## for

# Stable and Water-Tolerant Deep Eutectic Solvent from Biomass-Derived 5-Hydroxymethylfurfural (HMF) and Levulinic Acid

*Grazia Isa C. Righetti[a], Sara Rozas[b, c], Maria Enrica Di Pietro[a], Sara Santamaría[b], Sahar Nasrallah[d], Mirjana Minceva[d], Francesco Briatico Vangosa[a], Santiago Aparicio[b, e] and Andrea Mele[a,*]*

[a] Department of Chemistry, Materials and Chemical Engineering "G. Natta", Politecnico di Milano, 20133 Milano, Italy

[b] Department of Chemistry, University of Burgos, 09001 Burgos, Spain

[c] Centre for Cooperative Research on Alternative Energies (CIC energiGUNE), Basque Research and Technology Alliance (BRTA), Álava Technology Park, Albert Einstein 48, 01510 Vitoria-Gasteiz, Spain

[d] Biothermodynamics, TUM School of Life Sciences, Technical University of Munich, 85354 Freising, Germany

[e] International Research Centre in Critical Raw Materials-ICCRAM, University of Burgos, 09001 Burgos, Spain

*Corresponding author: andrea.mele@polimi.it (A. M.)

Number of pages: 33

Number of figures: 15

Number of tables: 12

# Materials and methods

*Water content, viscosity, density measurements and thermogravimetric analysis*

The water content was measured in triplicate using Karl Fisher titration method performed on an MKC-710 B instrument by KEM Kyoto Electronics.

Viscosity was measured in triplicate at 40°C, using an Anton Paar MCR502 rheometer with a cone-plate configuration (50mm diameter, 1° angle, and 99µm truncation). Temperature was controlled with a peltier plate and hood system (H-PTD – P-PTD 200). Shear rate was varied following a logarithmic ramp profile, ranging from 10 $s^{-1}$ to 100 $s^{-1}$, with data points collected at a density of 15 points per decade. Viscosity values were determined through the linear regression of the shear stress dependence on shear strain rate.

The density (ρ) of HMF:LEV mixtures was determined at different temperatures and molar ratios using a vibrating-tube densimeter (Anton Paar DMA 1001, uncertainty ±1 × $10^{-4}$ g $cm^{-3}$). Prior to the measurements, the instrument was calibrated by verifying the density of air and water at 293 K, ensuring that the obtained values were within the accepted tolerance range. After calibration, the samples were prepared for measurement. Each mixture was carefully introduced into the U-shaped cell of the densimeter with the aid of a syringe and a rubber tube, ensuring that the filling was uniform and that no air bubbles remained trapped inside the cell. The sample was injected through the inlet until it flowed out of the outlet, guaranteeing that the entire cell was filled. The temperature of the measurement cell was controlled by a Peltier unit, with a stability of ±0.01 K throughout the experiments. Before recording the measurements, the homogeneity of the sample inside the cell was visually confirmed using the built-in camera of the densimeter. Once these conditions were met, the density of the HMF:LEV mixtures was measured under the selected experimental conditions.

TGA analyses were performed with Mettler Toledo TGA2 instrument. Samples' weight ranged from 5 to 20 mg; as sample holders were used alumina crucibles. The analyses were carried out with a program that provides a single heating cycle from 30 °C to 500 °C at 20 °C/min under nitrogen or air atmosphere (50 ml/min).

The viscosity deviations were calculated using the following formula:

$$\Delta\eta = \eta - (x_{HMF}\eta_{HMF} + x_{LEV}\eta_{LEV}) \tag{1}$$

where $\eta$ is the mixture viscosity, $\eta_{HMF}$ and $\eta_{LEV}$ are the pure HMF and LEV viscosities, respectively and $x_{HMF}$ and $x_{LEV}$, the molar compositions of HMF and LEV in the mixture.

The excess molar volume is calculated using the following equation:

$$V^E = \frac{x_{HMF}M_{HMF} + x_{LEV}M_{LEV}}{\rho_{mix}} - \left(\frac{x_{HMF}M_{HMF}}{\rho_{HMF}} + \frac{x_{LEV}M_{LEV}}{\rho_{LEV}}\right) \tag{2}$$

where $\rho_{mix}$ stands for the density of the mixture, $\rho_{HMF}$ and $\rho_{LEV}$ are the pure HMF and LEV densities, respectively and $x_{HMF}$ and $x_{LEV}$ and $M_{HMF}$ and $M_{LEV}$, the molar compositions and molar weights of HMF and LEV, respectively.

*Differential scanning calorimetry*

DSC analysis was used to measure the melting properties of pure HMF and the SLE data for the HMF-LEV binary system. The DSC device (NETZSCH DSC 200 F3, Germany) was calibrated using six reference standards (adamantane, indium, tin, zinc, bismuth, and cesium chloride) at a heating rate of 5 K min$^{-1}$. Measurements were performed in an inert environment using nitrogen with a flow rate of 150 mL min$^{-1}$. Samples were weighed using a Sartorius analytical balance (Germany) with an uncertainty of ±0.01 mg.

Prior to analysis, the HMF was dried in a rotary evaporator at 298 K under vacuum (1 mbar), and LEV was preheated and used in its liquid state at room temperature. For the measurement of pure HMF, the DSC chamber was precooled to 273 K, followed by a heating cycle at a rate of 5 K min$^{-1}$ up to 323 K. The melting temperatures ($T_m$) and melting enthalpies ($\Delta h_m$) were obtained from the onset temperature and peak area, respectively. The DSC curve of pure HMF is shown in **Scheme S1**.

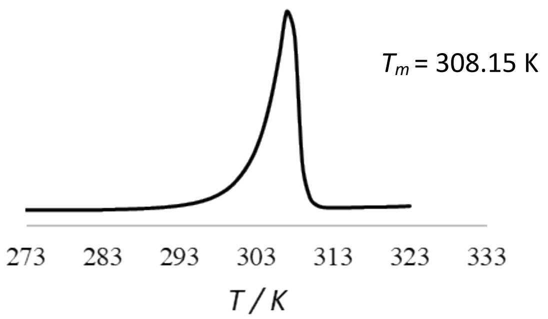


**Scheme S1**. DSC thermogram of pure HMF.

Binary HMF-LEV mixtures with different compositions ($x_{\mathrm{HMF}}$ = 0.12 to 0.91) were prepared, sealed in glass vials, and gently heated up to 310-320 K under continuous mixing until a clear liquid was formed. The samples were introduced in DSC crucible pans as a liquid in triplicate. The DSC pans were hermetically sealed and stored at 193 K to quench the liquid samples, then transferred and stored at 253 K for annealing for at least 2 months. For the binary mixtures, the samples were transferred from the 253 K freezer to the DSC using an isolated ice box. The DSC chamber was precooled to 243 K before introducing the sample. A cooling cycle at a rate of 5 K min$^{-1}$ down to 193 K was then performed, followed by a heating cycle at the same rate up to 323 K. The solidus and liquidus temperatures were determined as the onset of the first peak and the maximum of the second peak, respectively.

*Computational methods*

*(A) Thermodynamic Modeling for SLE*

The liquidus lines in the SLE phase diagrams can be calculated using eq. 3:

$$ln\ x_i\gamma_i = -\frac{\Delta h_{m,i}}{RT}\left(1-\frac{T}{T_{m,i}}\right)-\frac{\Delta c_{P,i}}{R}\left(1-\frac{T_{m,i}}{T}+ln\frac{T_{m,i}}{T}\right) \tag{3}$$

where $x_i$ and $\gamma_i$ are the mole fraction and activity coefficient of the component $i$ in the liquid phase, respectively; *T* is the temperature; $\Delta h_{\mathrm{m,i}}$ and $T_{\mathrm{m,i}}$ are the melting enthalpy and temperature of pure component $i$, respectively; $\Delta c_p$ is the difference between the constant pressure heat capacity of pure component $i$ in the solid and liquid states at $T_{\mathrm{m,i}}$; *R* is the universal gas constant. In many cases, the $\Delta c_p$ term has a minor influence on the solubility curve compared to the $\Delta h_{\mathrm{m,i}}$ term.[1] Thus, for the sake of simplicity, $\Delta c_p$ was not considered as a parameter in this study, and eq. 4 was used to calculate the solubility line:

$$\ln\ x_i\gamma_i = -\frac{\Delta \mathrm{h_{m,i}}}{\mathrm{RT}}\left(1-\frac{\mathrm{T}}{\mathrm{T_{m,i}}}\right) \tag{4}$$

The activity coefficients of the components in the liquid phase were calculated using the NRTL model[2] as follows:

$$\ln(\gamma_i) = \frac{\sum_{j=1}^{C}\tau_{ji}G_{ji}x_j}{\sum_{j=1}^{C}G_{ji}x_j}+\sum_{j=1}^{C}\frac{x_jG_{ij}}{\sum_{k=1}^{C}x_kG_{kj}}\left(\tau_{ij}-\frac{\sum_{k=1}^{C}x_k\tau_{kj}G_{kj}}{\sum_{k=1}^{C}x_kG_{kj}}\right) \tag{5}$$

$$G_{ij} = \exp(-\alpha_{ji}\tau_{ij}) \qquad G_{ji} = \exp(-\alpha_{ji}\tau_{ji}) \tag{6}$$

$$\tau_{ij} = \frac{(\mathrm{g}_{ij}-\mathrm{g}_{jj})}{RT} \qquad \tau_{ji} = \frac{(\mathrm{g}_{ji}-\mathrm{g}_{ii})}{RT} \tag{7}$$

where $(\mathrm{g}_{ij}-\mathrm{g}_{jj})$ and $(\mathrm{g}_{ji}-\mathrm{g}_{ii})$ are the binary interaction parameters and $\alpha_{ij}$ denotes the nonrandomness factor, assumed to equal 0.3. The binary interaction parameters were obtained by minimizing the following objective function:

$$\mathrm{OF(T)} = \sum_i^n\left(\frac{\left(T_i^{exp}-T_i^{cal}\right)^2}{n}\right)^{1/2} \tag{8}$$

where $T_i^{exp}$ and $T_i^{cal}$ are the experimental and calculated liquidus temperatures, respectively and $n$ is the number of data points. The discrepancy between the calculated liquidus temperatures ($T_i^{cal}$) and the experimentally measured temperatures ($T_i^{exp}$) was assessed by calculating the root-mean-square deviation (RMSD) as follows:

$$\mathrm{RMSD} = \sqrt{\frac{\sum_i^n\left(T_i^{exp}-T_i^{cal}\right)^2}{n}} \tag{9}$$

(B) *DFT and MD Simulation*

A conformational search for HMF and LEV molecules was carried out employing COSMOconf (COSMOtherm package, version 24.1.0) and DFT BP/TZVP calculations. Structures, relative energies and conformer probability distributions are shown in Figure S11. DFT calculations were performed using Orca software[3], with

B3LYP functional[4,5] , 6-311++G(d,p) basis set and D3 semiempirical method[6]. 1HMF:2LEV, 1HMF:1LEV, 2HMF:1LEV, 1HMF:1HMF and 1LEV:1LEV minimal clusters were constructed according to ABCluster[7] global optimization using xTB semiempirical method[8]. Among the six distinct minimal clusters initially evaluated for each HMF:LEV combination, the most thermodynamically stable configurations were selected for further analysis considering the addition of up to 5 $H_2O$ molecules.

The HMF:LEV minimal cluster interaction energies ($\Delta E$) were determined as the difference between the total energy of the cluster and the sum of the individual monomer energies. The HMF:LEV-water interaction energies ($E_{\text{int (HMF:LEV – water)}}$) were calculated as the difference between the total energy of the cluster and the sum of the HMF:LEV interaction energy and the water monomer energy. To mitigate the Basis Set Superposition Error, the counterpoise correction to the energy was applied.[9] The characterization of hydrogen bonding topology was conducted within the Quantum Theory of Atoms in Molecules (QTAIM) framework. Key intermolecular interactions were examined via Bond Critical Points (BCPs), based on electron density ($\rho_e$) and the Laplacian of electron density ($\nabla^2\rho_e$). Additionally, the optimized clusters were subjected to Non-Covalent Interaction (NCI) analysis.[10]

Phase equilibrium properties of the investigated HMF:LEV deep eutectic solvent were predicted using the COnductor-like Screening MOdel for Real Solvents (COSMO-RS) model,[11] using COSMOtherm. COSMO files for the individual species were generated from the optimized molecular structures with DFT (BP86/def-TZVP) calculations. The provided melting temperatures and fusion enthalpies of pure compounds were 308.20 K and 19.8 kJ mol$^{-1}$ for HMF[12] and 306.20 K and 9.22 kJ mol$^{-1}$ for LEV.[12] Four HMF conformers and two LEV conformers were employed, according to probability distribution values.

Molecular Dynamics (MD) simulations were carried out using MDynaMix v.5.3 software[13] and Merck Molecular Force Field,[14] as obtained from the SwissParam database.[15] Force field parameters with atomic charges assigned based on ChelpG DFT calculations[16] are summarized in Table S11. Inferred MD densities were compared with the experimentally measured (deviations of 0.75 to 2.91 %), confirming the reliability of MD simulations (Table S9). Pure HMF:LEV and HMF:LEV-water mixtures, with three different HMF:LEV molar ratios (1:2, 1:1 and 2:1), at 313 K and 283 and 313 K for the eutectic system (1HMF:2LEV), and 1 bar of pressure were considered for the simulations. Water content was considered based on experimental water percentage values, in order to replicate real samples moisture. Initial cubic simulation box configurations were built with Packmol software[17] and subjected to a two-stage procedure: equilibration run of 10 ns within the canonical ensemble (NVT) at 498 K, followed by a production phase of 30 ns under the isothermal-isobaric ensemble (NPT) at the specified temperature conditions. Temperature and pressure were controlled using the Nose-Hoover thermostat and barostat.[17]. The Tuckerman-Berne[18] double time-step algorithm, employing time increments of 3 femtoseconds (fs) for long-range interactions and 0.3 fs for short-range interactions was used. Electrostatic interactions were computed using the Ewald summation method[19] with a cut-off radius of 10 Å, while Lennard-Jones interactions were truncated at 10 Å. Cross-interaction parameters were determined based on the Lorentz-Berthelot mixing rules. Post-simulation trajectory analyses were performed using TRAVIS[20] and VMD[21] software packages.

*NMR measurements*

Pure mixtures were transferred to a 5 mm NMR tube, equipped with a coaxial insert containing deuterated dimethyl sulfoxide (DMSO-d6). NMR measurements were performed at 313 K without sample spinning with a Bruker NEO 500 console (11.74 T) equipped with a direct observe BBFO (broadband including fluorine) iProbe and a variable-temperature unit ($^1$H resonance frequency of 500.13 MHz). The instrument was carefully tuned, shimmed, and the 90 pulses calibrated. $^1$H self-diffusion coefficients were measured by pulsed field gradient (PFG) NMR experiments by applying sine-shaped pulsed magnetic field gradients along the z-direction up to a maximum strength of G = 48.15 G $cm^{-1}$. The diffusion experiments were performed using the bipolar pulse longitudinal eddy current delay (BPP-LED) pulse sequence. All experiments were carried out using 16384 points in the F2 dimension, over a spectral width of 15 ppm. The relaxation delay was set to 10 s. The pulse gradients were incremented from 2 to 95% of the maximum gradient strength in a linear ramp with 32 steps. For each DOSY experiment, the duration of the magnetic field pulse gradients (δ) and the diffusion times (Δ) were optimized to obtain, where possible, 95% signal attenuation for the slowest diffusion species in the last step experiment. δ values were in the 2.0−6.0 ms range, while Δ values were 0.2−0.8 s long. The baselines of all arrayed spectra were corrected prior to processing the data. Data were processed using an exponential filter in F2 dimension (LB = 0.3 Hz), and integrals were used in calculating relaxation times. The diffusion decays obtained by PFG-NMR were analyzed using the Stejskal-Tanner equation implemented in the Bruker T1/T2 module of TopSpin[22,23] :

$$\frac{I}{I_0} = exp\left(-\gamma^2 g^2 \delta^2 D \left(\Delta - \frac{\delta}{3}\right)\right) \qquad (10)$$

where $I$ and $I_0$ are the echo intensity with and without field gradient, $\gamma$ is the gyromagnetic ratio, $g$ and $\delta$ are the gradient strength and duration, and Δ is the diffusion time.

Far above the liquidus temperature the atomic quantity $D$ and the macroscopic property $\eta$ are connected by the Stokes-Einstein (SE) relation[24]

$$D = \frac{kT}{6\eta\pi r} \qquad (11)$$

where $k$ is the Boltzmann constant, $T$ the absolute temperature, $r$ the apparent hydrodynamic radius of the particle.

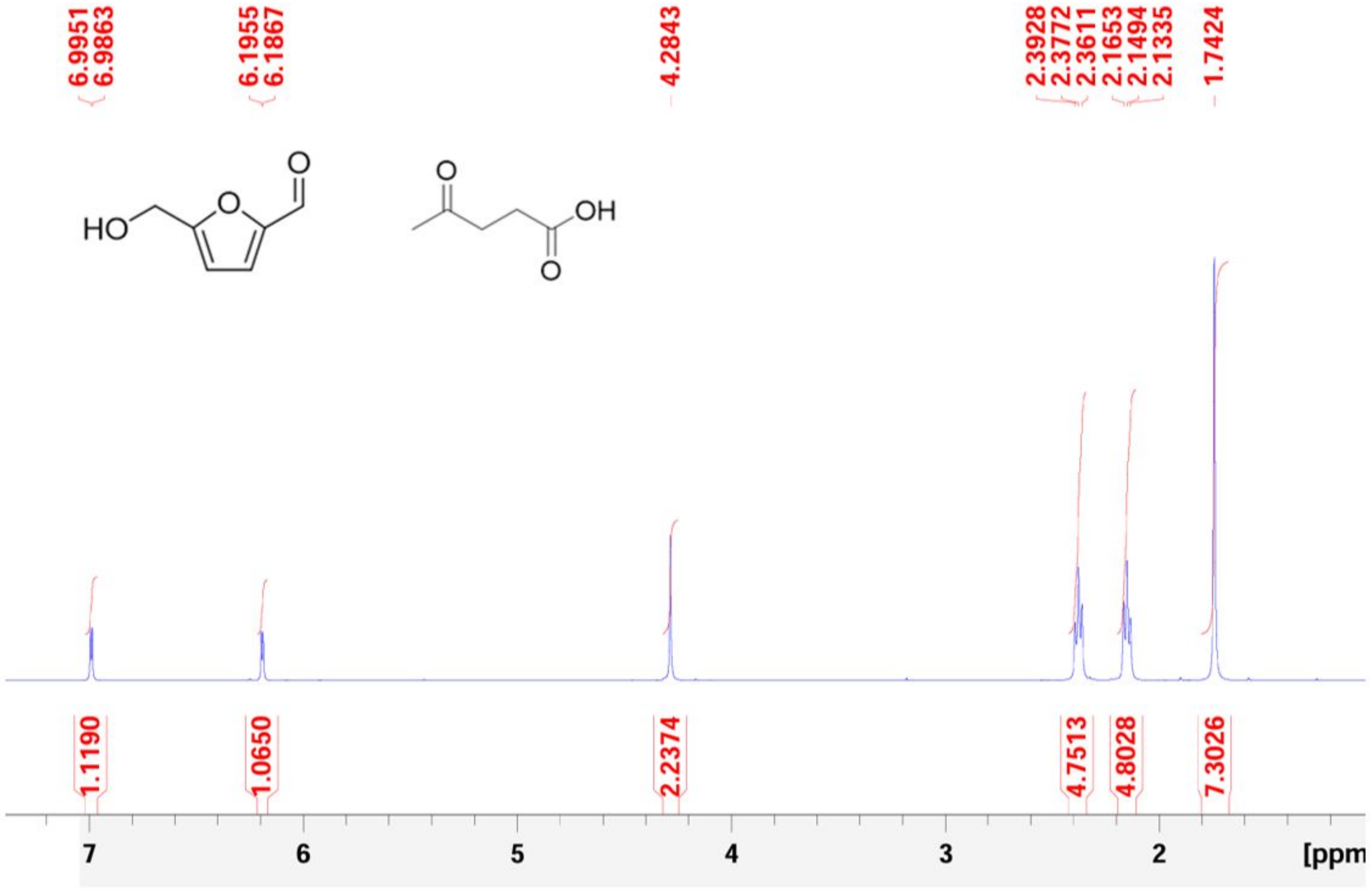


**Figure S1** $^1$H-NMR of the pure 1HMF-2LEV DES (DMSO-d6 coaxial inset), δ (ppm): 9.20 (s, 1H, -CHO), 7.1-1.09 (d, 1H, -CH- (2), J = 3.4 Hz), 6.31-6.30 (d, 1H, -CH- (3), J = 3.4 Hz), 4.41 (s, 1H, -$CH_2$OH), 2.51-2.48 (t, 2H, -CO-$CH_2$- (2'), J = 6.3 Hz), 2.28-2.26 (t, 2H, --$CH_2$-$CH_2$- (3'), J = 6.3 Hz), 1.86 (s, 3H, $CH_3$ (1')-CO-)

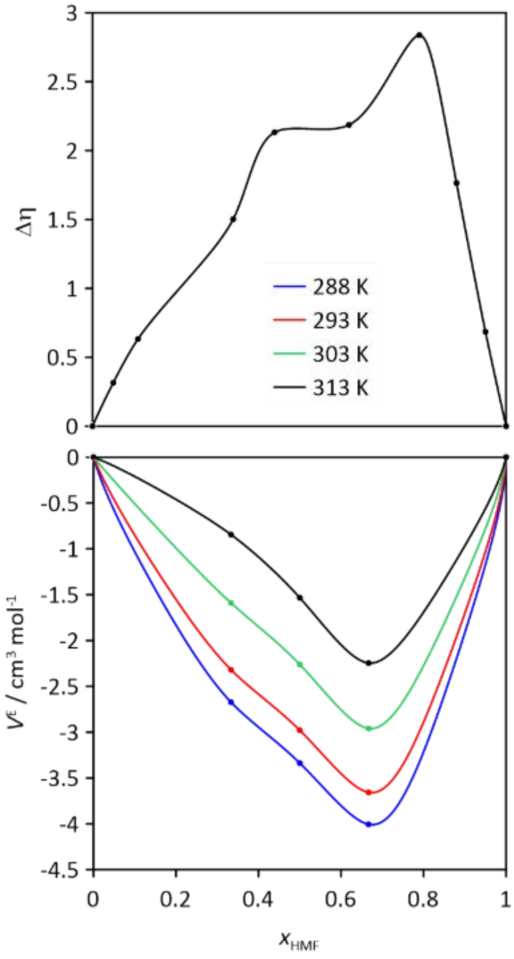


**Figure S2** Viscosity deviation, $\Delta\eta$, (top-panel) and excess molar volume, $V^E$, (bottom panel) as a function of HMF molar fraction for HMF : LEV mixtures at different concentrations.

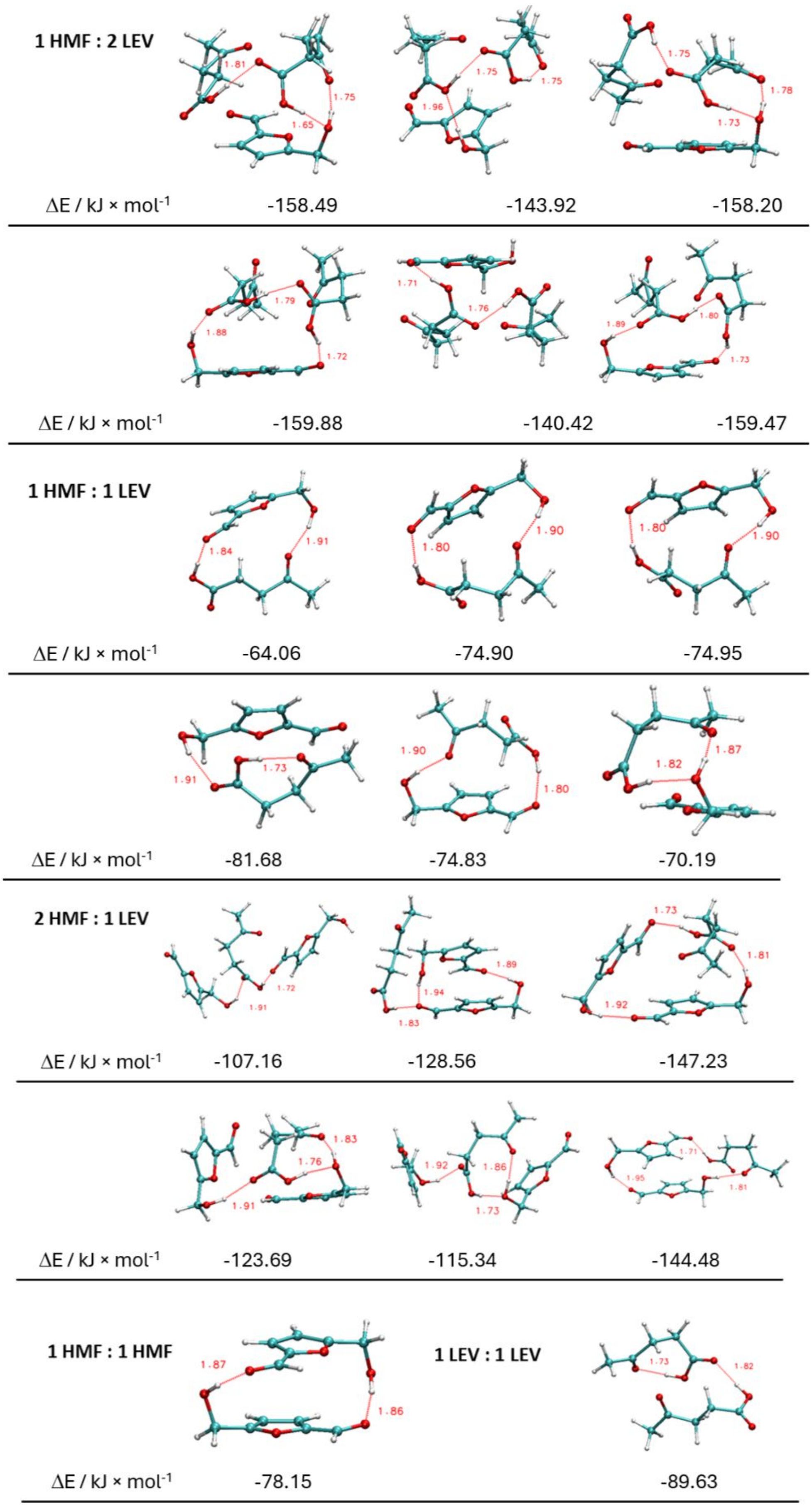


**Figure S23** DFT optimized structures, interaction energies, Δ*E*, and relevant interatomic distances of 1HMF:2LEV, 1HMF:1LEV, 2HMF:1LEV, 1HMF:1HMF and 1LEV:1LEV minimal clusters.

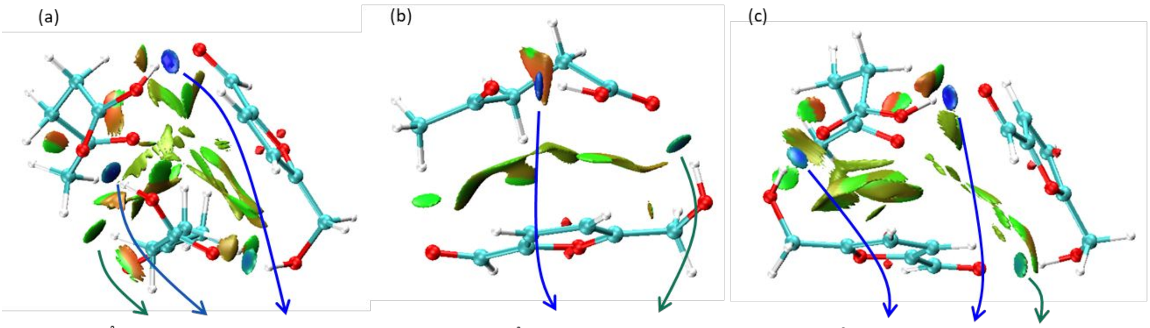


(a)

| | | | |
|---|---|---|---|
| $d_{H\text{-bond}}$ / Å | 1.89 | 1.80 | 1.73 |
| $\phi_{H\text{-bond}}$ / ° | 161.03 | 169.73 | 154.5 |
| $\rho$ / u.a. | 0.03 | 0.04 | 0.04 |
| $\nabla^2\rho$ / u.a. | 0.10 | 0.11 | 0.13 |

(b)

| | | |
|---|---|---|
| $d_{H\text{-bond}}$ / Å | 1.73 | 1.91 |
| $\phi_{H\text{-bond}}$ / ° | 156.68 | 153.39 |
| $\rho$ / u.a. | 0.04 | 0.03 |
| $\nabla^2\rho$ / u.a. | 0.13 | 0.10 |

(c)

| | | | |
|---|---|---|---|
| $d_{H\text{-bond}}$ / Å | 1.81 | 1.73 | 1.92 |
| $\phi_{H\text{-bond}}$ / ° | 162.88 | 159.92 | 148.21 |
| $\rho$ / u.a. | 0.03 | 0.04 | 0.02 |
| $\nabla^2\rho$ / u.a. | 0.12 | 0.13 | 0.10 |

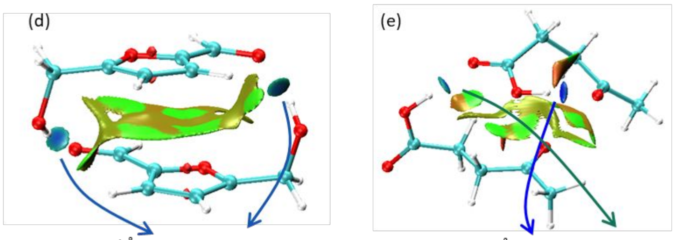


(d)

| | | |
|---|---|---|
| $d_{H\text{-bond}}$ / Å | 1.86 | 1.87 |
| $\phi_{H\text{-bond}}$ / ° | 169.29 | 159.49 |
| $\rho$ / u.a. | 0.03 | 0.03 |
| $\nabla^2\rho$ / u.a. | 0.10 | 0.10 |

(e)

| | | |
|---|---|---|
| $d_{H\text{-bond}}$ / Å | 1.73 | 1.82 |
| $\phi_{H\text{-bond}}$ / ° | 154.95 | 173.90 |
| $\rho$ / u.a. | 0.04 | 0.03 |
| $\nabla^2\rho$ / u.a. | 0.13 | 0.11 |

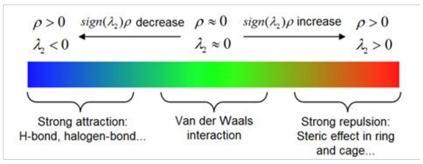


**Figure S4** DFT analysis of (a) 1HMF:2LEV, (b) 1HMF:1LEV, (c) 2HMF:1LEV, (d) 1HMF:1HMF and (e) 1LEV:1 LEV most stable configurations, showing hydrogen bonds distance and angle, QTAIM analysis at the identified BCP and NCI analysis.

COSMO-RS computed $\sigma$-surfaces and $\sigma$-profiles (**Figure S5**) clearly underline a complex hydrogen bond donor/acceptor character of both HMF and LEV. The latter illustrate the charge distribution probability along the molecular surface. While $\sigma$-surfaces and $\sigma$-profiles of HMF and LEV exhibit similar donor character (blue and red curves, respectively), LEV displays stronger acceptor character, consistent with its chemical structure. Furthermore, both HMF and LEV species exhibit large non-polar interaction regions, meaning high dispersion interactions probability. The stronger acceptor character of LEV, compared to HMF, along with the strong donor capacity of water aligns with DFT results (1HMF:2LEV-water strong interactions through LEV acceptor sites, Table S5).

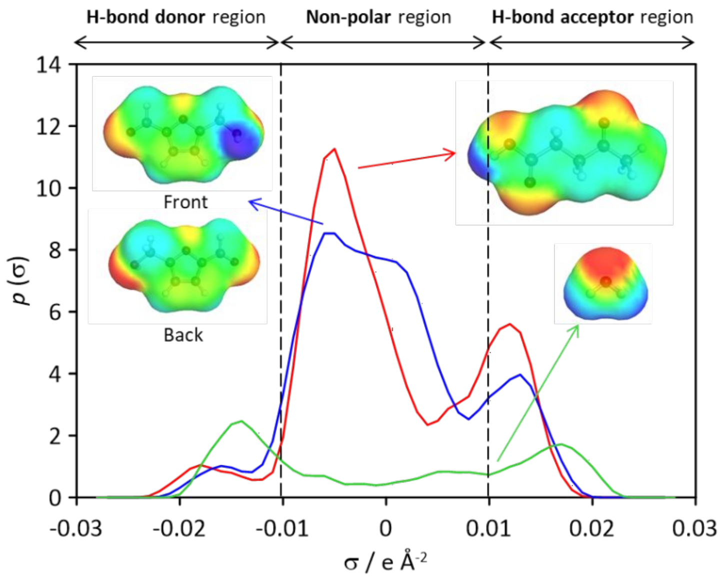


**Figure S5** $\sigma$-profiles and $\sigma$-surfaces calculated from COSMO-RS for HMF (blue), LEV (red) and water (green) from DFT geometry optimization calculations

The HMF:LEV-water interaction energy per water molecule, $E_{HMF:LEV\text{-}water}$ / $n_{water}$, was calculated as the difference between the total energy of the cluster and the sum of the HMF:LEV interaction energy ($\Delta E$ from FigureS2) and the water monomer energy divided by the number of water molecules. Therefore, with this value we quantify the interaction energy coming only from the water molecules.

$$E_{HMF:LEV-water} = E_{total} - E_{int(HMF:LEV)} - n \times E_{water}$$

where $E_{total}$ is the total energy of the HMF:LEV-water cluster, $E_{int(HMF:LEV)}$ is the interaction energy originated by the clean HMF-LEV clusters and $n$ is the number of water molecules. $E_{HMF:LEV\text{-}water}$ / $n_{water}$ values aim at providing a quantitative parameter of the water addition effect, which may be additive, synergistic or antagonistic.

Trigonal bipyramids (tbp) water pentamer, as the one identified in 1HMF:2LEV system (**Figure S6**) exhibits higher hydrogen donor ability as already reported by Wales et al.[24]. This fact, combined with LEV available acceptor sites within the 1:2 cluster, enhances HMF:LEV-water hydrogen bonding interaction.

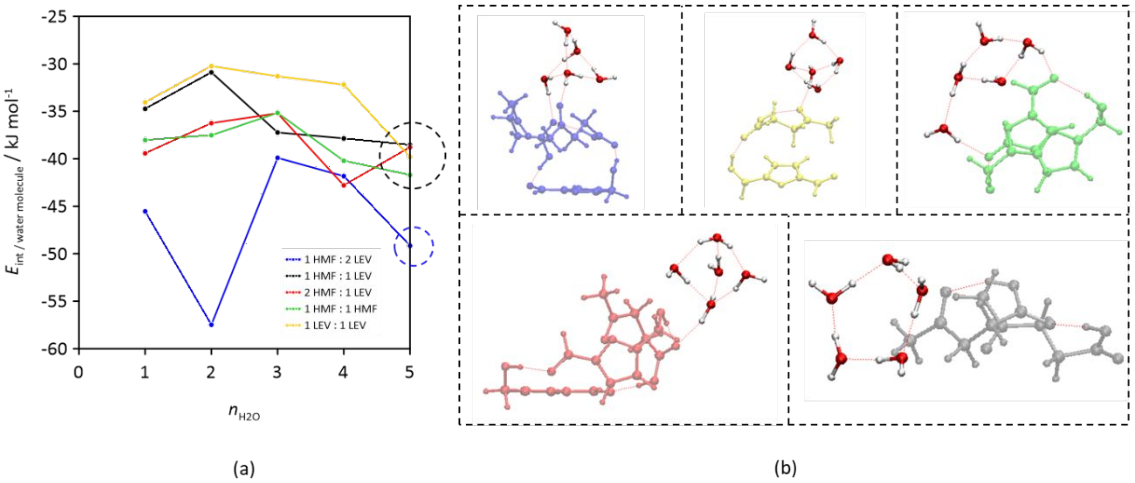


**Figure S6** (a) HMF:LEV-water interaction energy per water molecule ($E_{HMF:LEV\text{-}water}$ / n $_{water}$), (b) optimized geometry configurations from DFT calculations for all the considered clusters configurations with n $_{water}$ = 5.

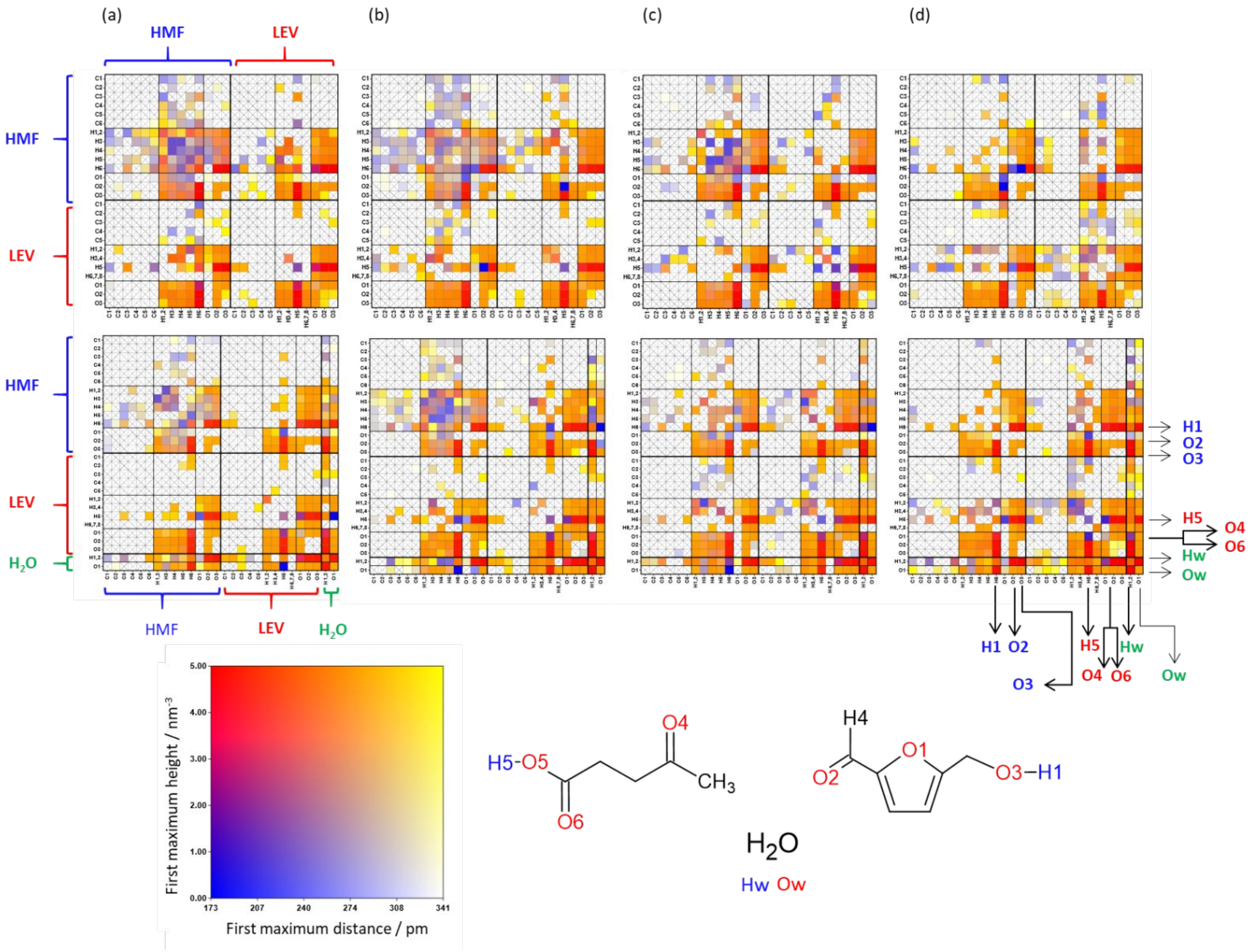


**Figure S7** Connection matrix, *cmat*, analysis of the reported pure HMF:LEV (top panels) and HMF:LEV + water (bottom panels) systems at (a) 1:2 and 283 K, (b) 1:2 and 313 K, (c) 1:1 and 313 K, and (d) 2:1 and 313 K HMF:LEV molar ratio and temperature conditions from molecular dynamics simulations. The color in each square represents both the intensity and distance of the first maximum in the corresponding *rdf*. Atom labelling is shown for comprehension purposes**.**

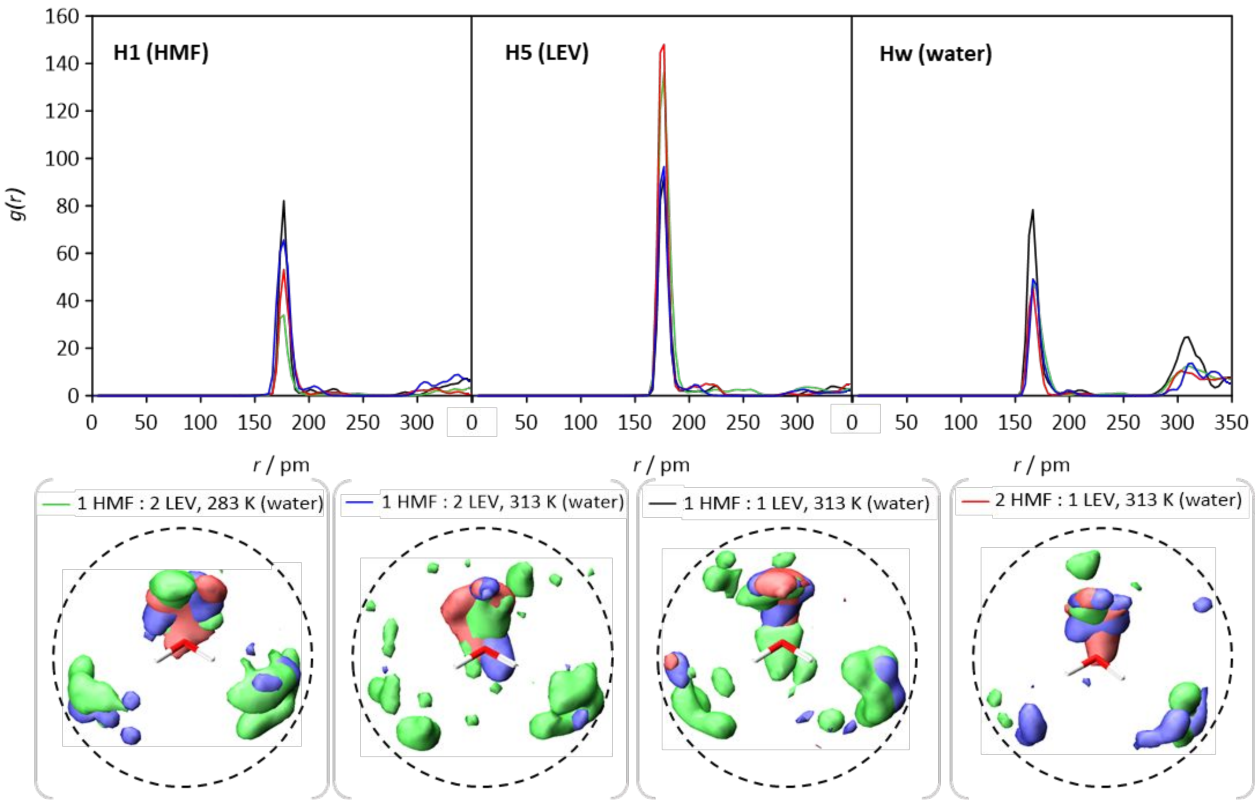


**Figure S8** Site – site radial distribution functions, *g(r)*, for the pairs considering water(Ow) site and hydrogen HMF(H1), LEV(H5) and water(Hw) donor -atoms labelling as in Figure 1 - for the considered HMF:LEV + water systems from molecular dynamics simulations at the considered HMF:LEV molar ratios and temperature conditions. Figures show spatial distribution functions, *sdf's*, of the considered systems. Isosurface color code: blue for HMF, red for LEV and green for water. Isusurface value: 0.25.

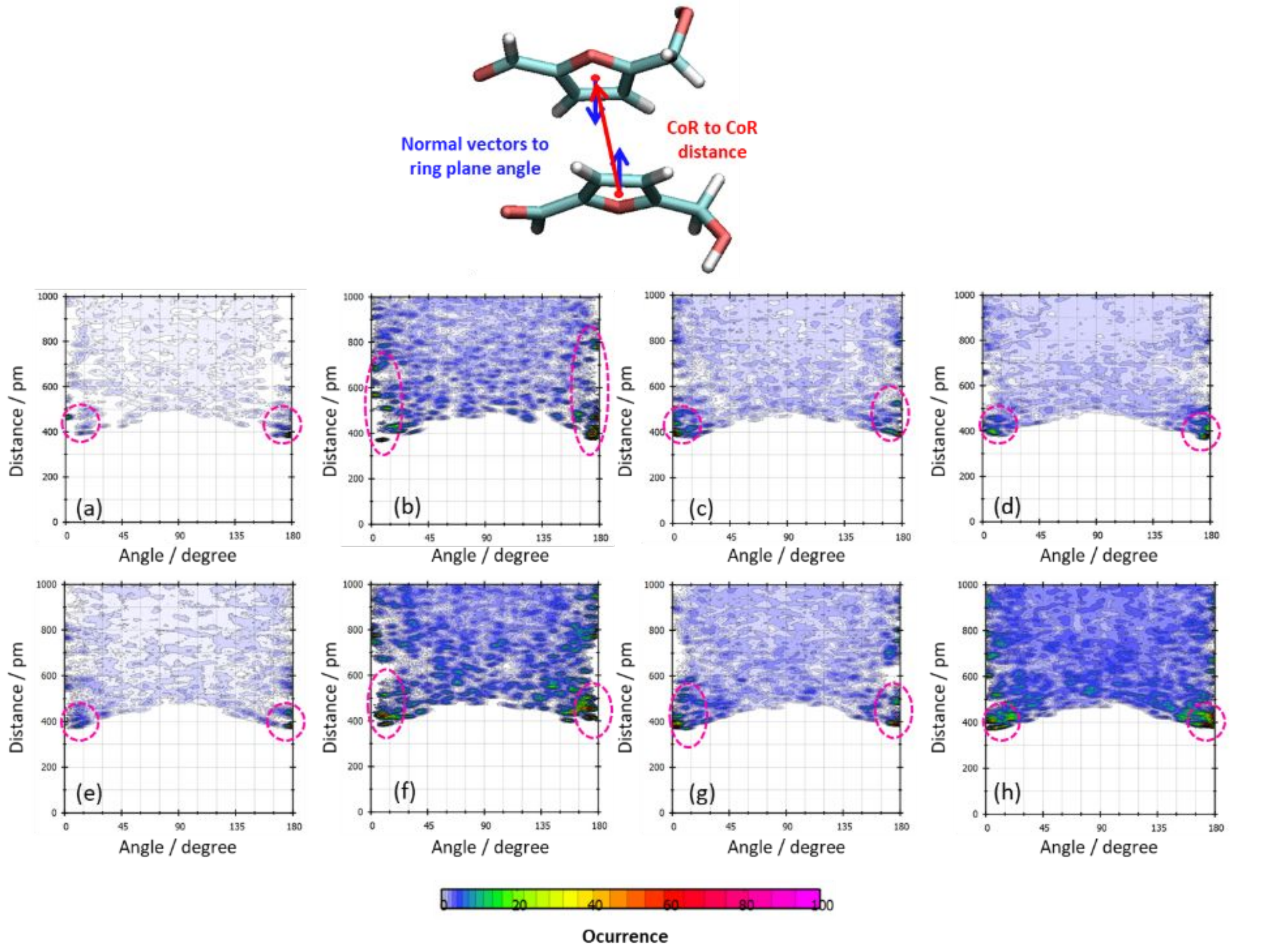


**Figure S9** Combined distribution functions, *cdf*, for the the angle formed between the normal vectors of HMF ring plane and the CoR-CoR distance, in (a, b) 1 HMF : 2 LEV, (c) 1 HMF : 1 LEV and (d) 2 HMF : 1 LEV mixtures; and (e, f) 1 HMF : 2 LEV + water, (g) 1 HMF : 1 LEV + water and (h) 2 HMF : 1 LEV + water mixtures. Results from MD simulations at (a, b, d, e, f, h) 313 K and (c, g) 283 K and 1 bar.

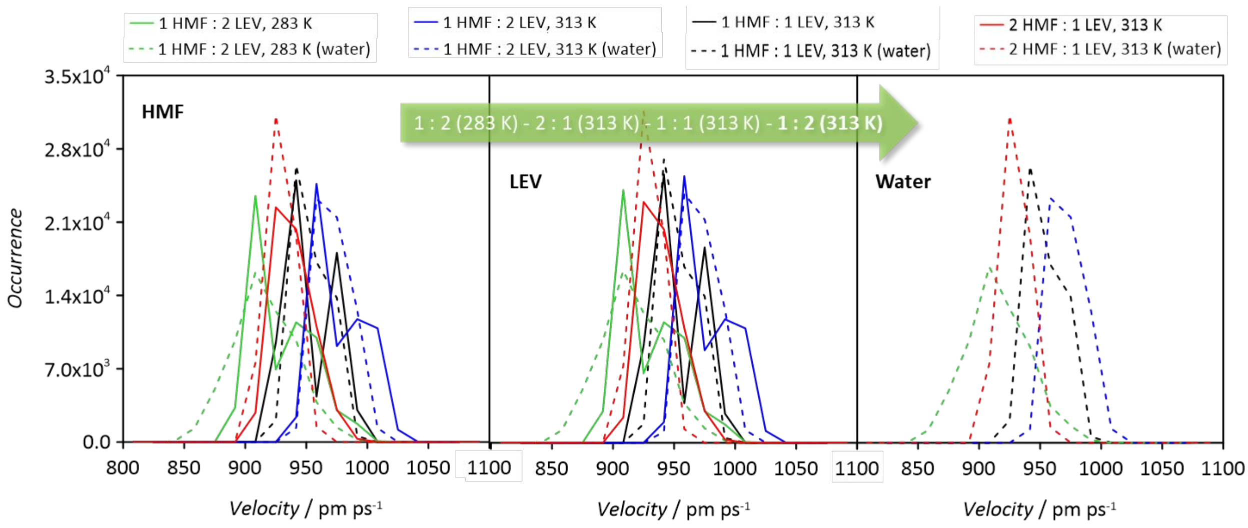


**Figure S10** Velocity distribution functions, *vdf's*, of HMF, LEV and water molecules on the considered HMF:LEV and HMF:LEV + water mixtures from molecular dynamics simulations at the considered HMF:LEV molar ratios and temperature conditions.

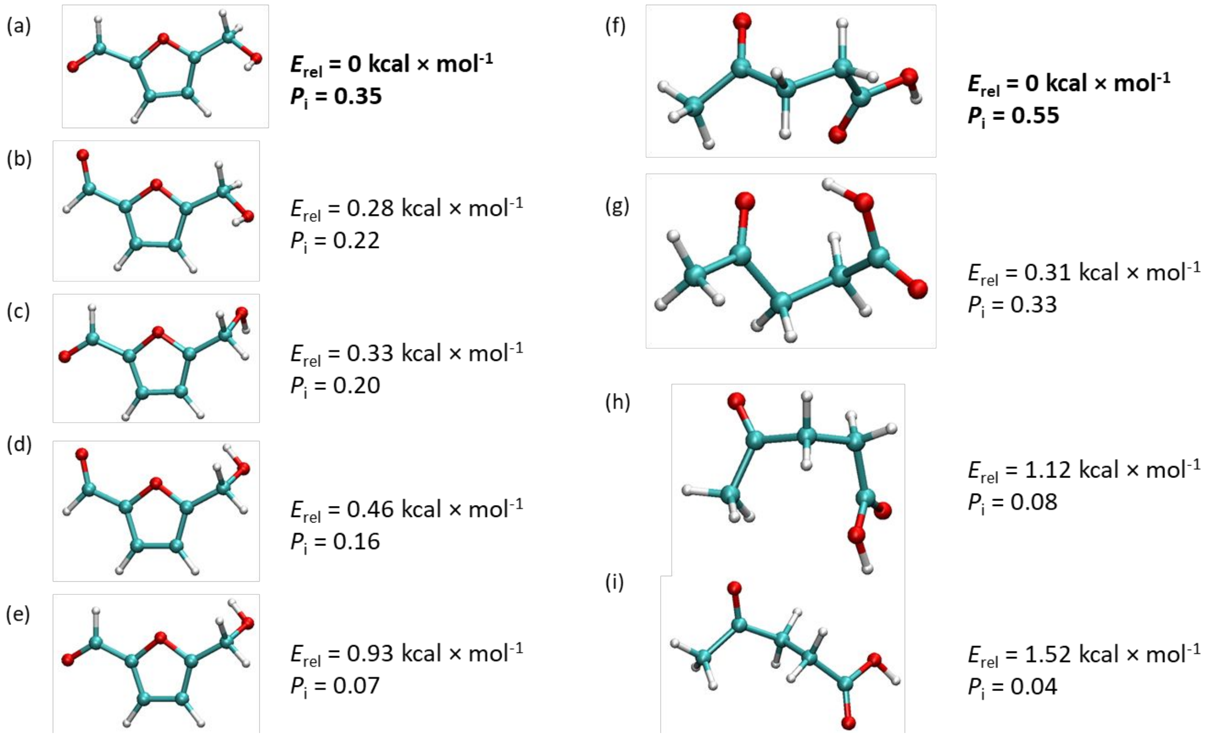


**Figure S11** Structure, relative energy, $E_{rel}$, and Boltzmann probability distribution, $P_i$, of (a-e) HMF conformers and (f-i) LEV conformers from COSMOconf conformational search employing DFT BP86/def-TZVP method.

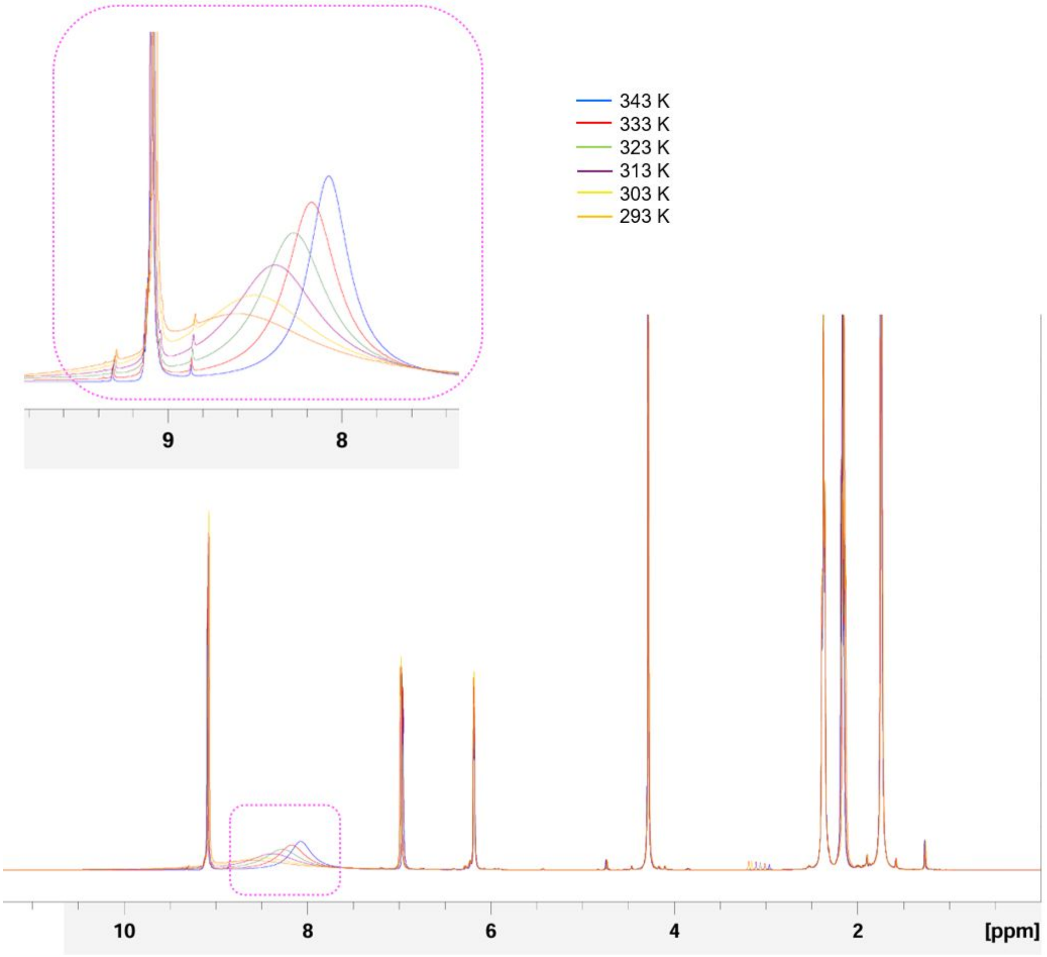


**Figure S12** Overlapped $^1$H-NMR spectra of 1HMF-2LEV – eutectic composition – at different temperatures, the inset graph shows a zoom-in of the average -OH signal detected

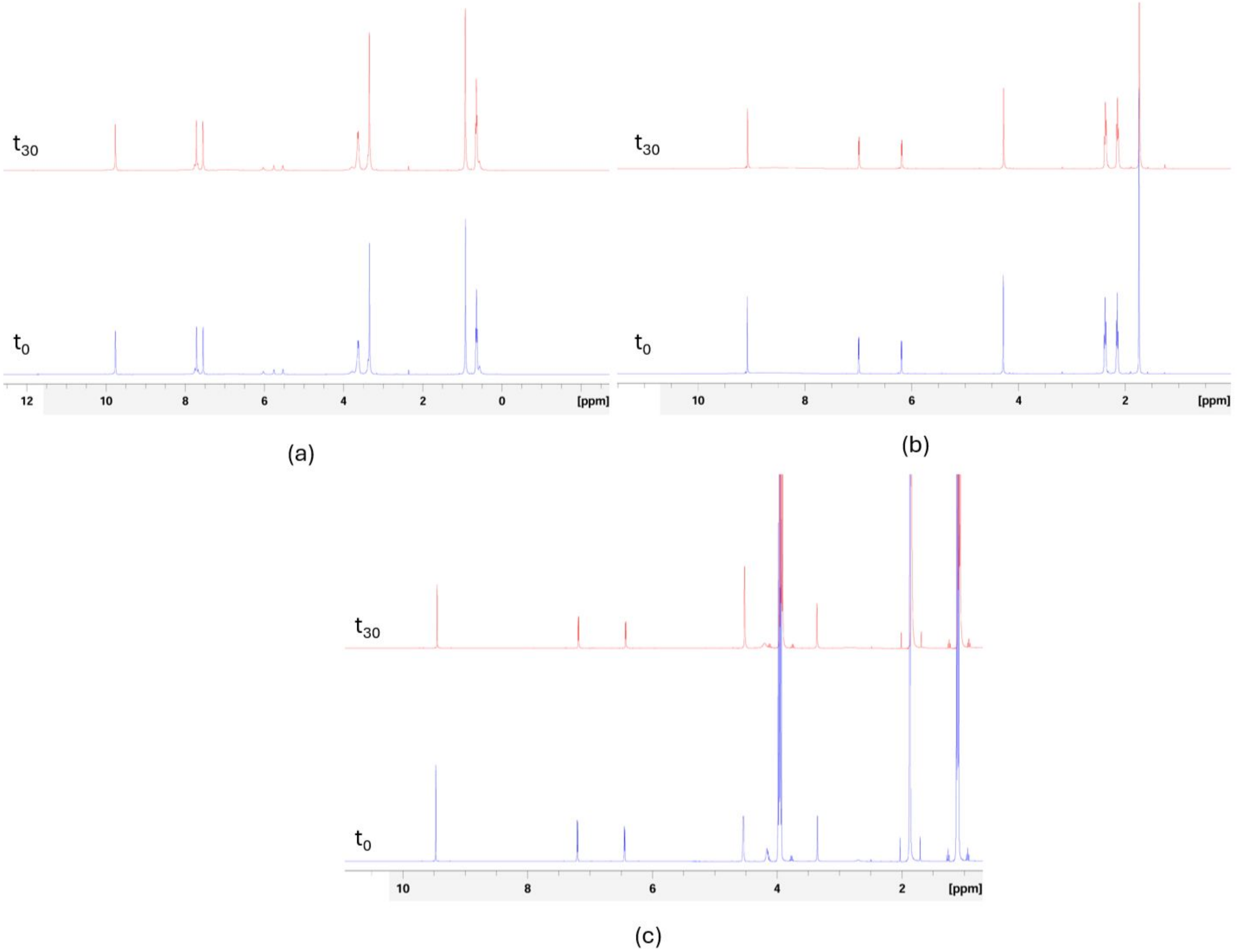


**Figure S13** Stability check of (a) HMF in 1-ethyl-3-methyl imidazolium acetate (5% wt solution), (b) neat HMF-LEV DES and (c) HMF in ethyl acetate (5% wt solution), $t_0$ = freshly prepared mixture, $t_{30}$ = same mixture after 30 days at room temperature

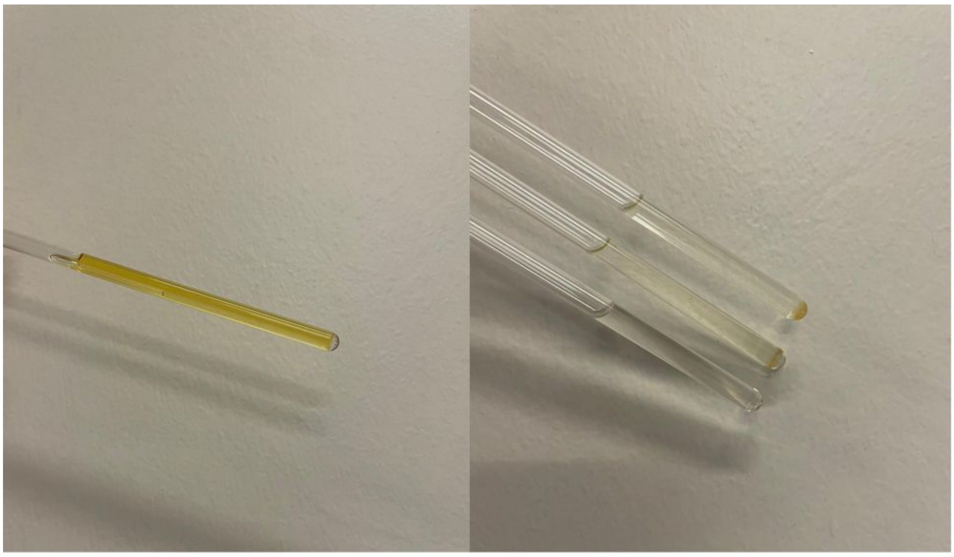

(a) (b)

**Figure S14** Physical appearance of (a) HMF-LEV DES and (b) HMF in AcOEt – 1%, 5% and 10% wt from bottom to top

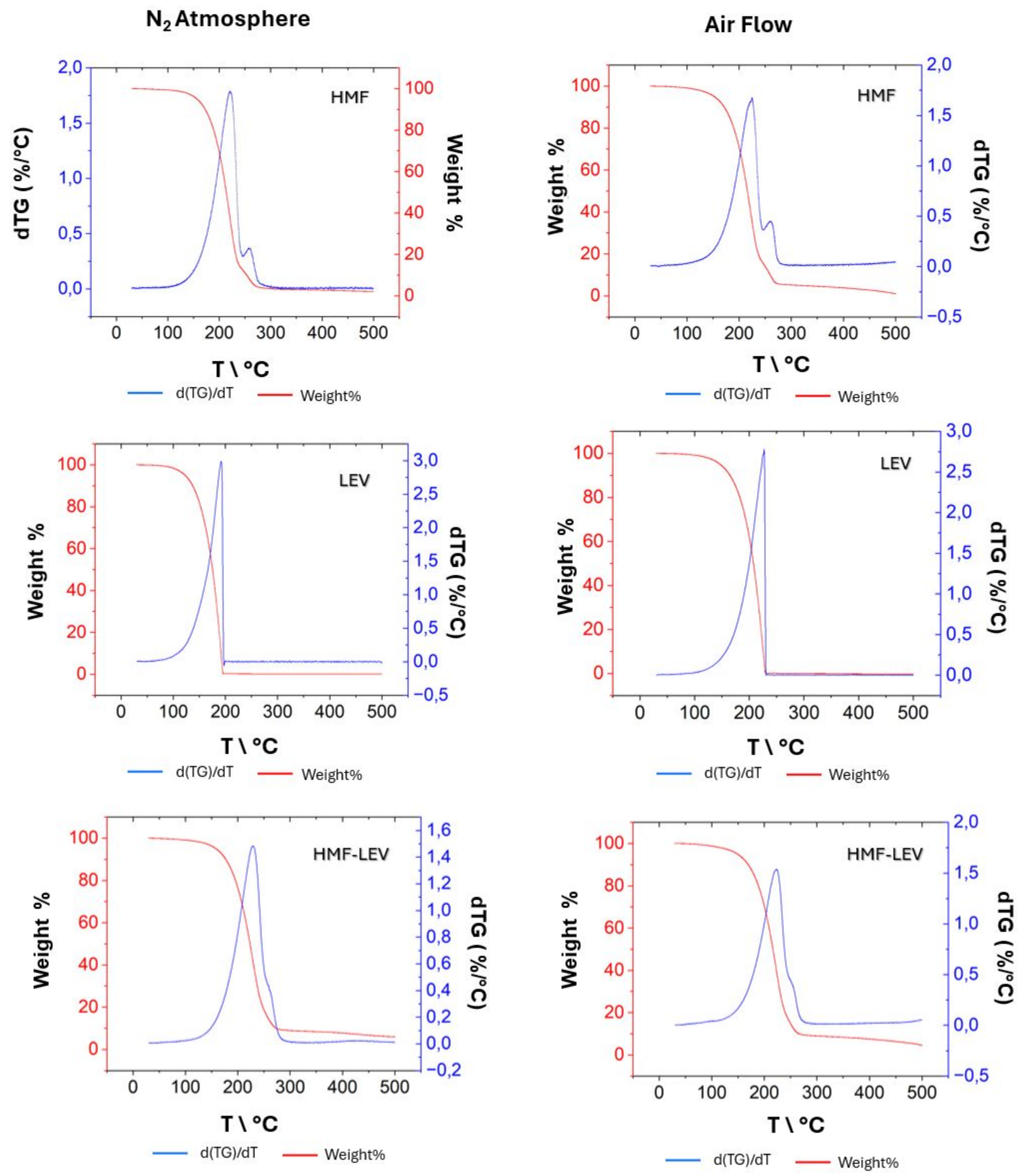


**Figure S15** TGA and dTGA of pure components and eutectic mixture under $N_2$ and air flow

**Table S1** Melting temperature ($T_m$) and enthalpy ($\Delta h_m$) of the pure HMF and LEV.

| **Compound** | **$T_m$/K** | | **$\Delta h_m$/ kJ mol$^{-1}$** | |
|---|---|---|---|---|
| | This work | Literature[12] | This work | Literature[12] |
| **HMF** | 308.15 ± 0.09 | 308.5 | 19.35 ± 0.22 | 19.8 |
| **LEV** | - | 306.2 | - | 9.22 |

**Table S2** Solid–liquid equilibria (SLE) data of HMF-LEV binary system

| $x_{HMF}$ | $T^{liq}/K$ | $T_e/K$ |
|---|---|---|
| 0.91 | 304.12 | 257.68 |
| 0.82 | 297.25 | 258.68 |
| 0.74 | 292.32 | 257.55 |
| 0.70 | 291.92 | 263.45 |
| 0.63 | - | 262.98 |
| 0.54 | - | 264.88 |
| 0.43 | - | 265.18 |
| 0.38 | - | 263.95 |
| 0.33 | - | 264.12 |
| 0.22 | 283.28 | 263.58 |
| 0.12 | 288.40 | 261.70 |

**Table S3** NRTL model binary interaction parameters along with the RMSD and infinite dilution activity coefficient ($\ln \gamma_i^\infty$ ) of components (HMF=1 and LIV=2) calculated at 298.1 K. The calculated eutectic compositions ($x_{\mathrm{HMF},e}^{NRTL}$) and eutectic temperatures ( $T_e^{NRTL}$) are presented together with the RMSD values. The experimental glass transition composition ($x_{\mathrm{HMF},g}^{exp}$) and temperature ($T_g^{exp}$) are also included for comparison. The non-randomness factor $\alpha_{12}$ = $\alpha_{21}$ is set to 0.3.

| **HMF (1) - LEV (2)** | |
|---|---|
| $(g_{12} - g_{22})$/ kJ mol$^{-1}$ | -0.1392 |
| $(g_{21} - g_{11})$/ kJ mol$^{-1}$ | -2.5219 |
| $\ln \gamma_1^\infty$ (-) | -1.0744 |
| $\ln \gamma_2^\infty$ (-) | -1.4366 |
| RMSD | 1.4615 |
| $x_{\mathrm{HMF},e}^{NRTL}$ (-) | 0.39 |
| $T_e^{NRTL}$ / K | 258.0 |
| $x_{\mathrm{HMF},g}^{exp}$ (-) | 0.38 |
| $T_g^{exp}$ / K | 241.8 |

**Table S4** Water content and viscosity measurements for the HMF-LA mixtures

| **Entry** | **$\chi_{HMF}$** | **$\chi_{LA}$** | **Water content (*wt. %*)** | **Viscosity (mPa × s)** |
|---|---|---|---|---|
| **1** | 0 | 1 | 0.42 | 15.5 |
| **2** | 0.05 | 0.95 | 0.29 | 15.8 |
| **3** | 0.11 | 0.89 | 0.34 | 16.1 |
| **4** | 0.34 | 0.66 | 1.17 | 16.9 |
| **5** | 0.44 | 0.56 | 1.12 | 17.5 |
| **6** | 0.62 | 0.38 | 1.02 | 17.5 |
| **7** | 0.79 | 0.21 | 1.04 | 18.1 |
| **8** | 0.88 | 0.12 | 0.75 | 17.0 |
| **9** | 0.95 | 0.05 | 0.71 | 15.9 |
| **10** | 1 | 0 | 0.88 | 15.2 |

**Table S5** DFT analysis of hydrogen bonds in 1HMF:2LEV, 1HMF:1LEV, 2HMF:1LEV, 1HMF:1 HMF and 1LEV:1LEV + *n* water molecules (n = 1, 2, 3, 4, 5) minimal clusters considering interaction energy ($E_{int}$) as well as geometrical and topological (QTAIM) parameters. Parenthesized data show values of $E_{int}$ per water molecule. Red shaded cells refer to starting HMF:LEV clusters, as provided in FigureS4.

| | | Geometry parameters | | | QTAIM | |
|---|---|---|---|---|---|---|
| $n \times H_2O$ | $E_{int\ (HMF\ :\ LEV\ –\ water)}$ / kJ mol$^{-1}$ | $H_{bond}$ (H---O) | $d_{H\text{-}bond}$ / Å | $\phi_{H\text{-}bond}$ / ° | $\rho$ / u.a. | $\nabla^2\rho$ / u.a. |
| | | **1HMF:2LEV** | | | | |
| 0 | $\Delta E$ = -159.47 kJ mol$^{-1}$ | LEV1(H5)-HMF(O2) | 1.73 | 154.5 | 0.04 | 0.13 |
| | | LEV2(H5)-LEV1(O6) | 1.8 | 169.73 | 0.04 | 0.11 |
| | | HMF(H1)-LEV2(O6) | 1.89 | 161.03 | 0.03 | 0.10 |
| 1 | **-45.54**<br>HMF(H1)-LEV2(O6) become extinct | LEV1(H5)-HMF(O2) | 1.67 | 161.46 | 0.05 | 0.14 |
| | | LEV2(H5)-LEV1(O6) | 1.73 | 171.29 | 0.04 | 0.13 |
| | | HMF(H1)-water(Ow) | 1.78 | 168.22 | 0.04 | 0.13 |
| | | Water(Hw)-LEV(O6) | 1.74 | 171.62 | 0.04 | 0.13 |
| 2 | -114.96 (**-57.48**)<br>HMF(H1)-LEV2(O6) become extinct | LEV1(H5)-HMF(O2) | 1.66 | 160.48 | 0.05 | 0.14 |
| | | LEV2(H5)-LEV1(O6) | 1.7 | 175.03 | 0.04 | 0.13 |
| | | HMF(H1)-water2(Ow) | 1.77 | 172.66 | 0.04 | 0.12 |
| | | Water1(Hw)-LEV2(O6) | 1.84 | 152.38 | 0.02 | 0.09 |
| | | Water1(Hw)-LEV2(O6) | 1.98 | 147.71 | 0.03 | 0.11 |
| 3 | -119.72 (**-39.90**)<br>HMF(H1)-LEV2(O6) become extinct | LEV1(H5)-HMF(O2) | 1.63 | 162.88 | 0.05 | 0.14 |
| | | LEV2(H5)-LEV1(O6) | 1.72 | 176.31 | 0.04 | 0.13 |
| | | HMF(H1)-water2(Ow) | 1.78 | 171.86 | 0.04 | 0.12 |
| | | Water1(Hw)-LEV2(O6) | 1.85 | 151.29 | 0.03 | 0.11 |
| | | Water1(Hw)-LEV2(O6) | 1.96 | 149.19 | 0.02 | 0.09 |
| | | Water3(Hw)-LEV1(O6) | 1.87 | 162.89 | 0.03 | 0.11 |
| 4 | -167.33 (**-41.83**) | LEV1(H5)-HMF(O2) | 1.7 | 158.06 | 0.04 | 0.13 |
| | | LEV2(H5)-LEV1(O6) | 1.81 | 172.07 | 0.03 | 0.11 |
| | | HMF(H1)-LEV2(O6) | 1.86 | 162.94 | 0.03 | 0.11 |
| | | Water1(Hw)-LEV2(O4) | 1.91 | 151.43 | 0.02 | 0.10 |
| | | Water2(Hw)-LEV1(O6) | 1.86 | 171.22 | 0.03 | 0.11 |
| 5 | -245.80 (**-49.16**) | LEV1(H5)-HMF(O2) | 1.7 | 155.77 | 0.04 | 0.13 |
| | | LEV2(H5)-LEV1(O6) | 1.85 | 167.46 | 0.03 | 0.10 |
| | | HMF(H1)-LEV2(O6) | 1.88 | 161.06 | 0.03 | 0.10 |
| | | Water1(Hw)-LEV1(O6) | 1.91 | 168.86 | 0.03 | 0.10 |
| | | Water2(Hw)-LEV2(O4) | 1.85 | 168.75 | 0.03 | 0.11 |
| | | **1HMF:1LEV** | | | | |
| 0 | $\Delta E$ = -81.68 kJ mol$^{-1}$ | LEV(H5)-LEV(O4) | 1.73 | 156.68 | 0.04 | 0.13 |
| | | HMF(H1)-LEV(O6) | 1.91 | 153.39 | 0.03 | 0.10 |
| 1 | **-34.74** | LEV(H5)-LEV(O4) | 1.68 | 162.91 | 0.04 | 0.13 |
| | | HMF(H1)-LEV(O6) | 1.92 | 151.77 | 0.03 | 0.10 |
| | | Water(Hw)-HMF(O2) | 1.8 | 168.99 | 0.03 | 0.10 |
| 2 | -61.79 (**-30.9**) | LEV(H5)-LEV(O4) | 1.72 | 158.43 | 0.04 | 0.13 |
| | | HMF(H1)-LEV(O6) | 1.9 | 154.99 | 0.03 | 0.10 |
| | | Water1(Hw)-LEV(O4) | 1.91 | 148.9 | 0.03 | 0.10 |
| 3 | -111.70 (**-37.23**) | LEV(H5)-LEV(O4) | 1.71 | 157.3 | 0.05 | 0.13 |
| | | HMF(H1)-LEV(O6) | 1.91 | 153.14 | 0.03 | 0.10 |
| | | Water1(Hw)-HMF(O2) | 1.87 | 161.14 | 0.03 | 0.10 |
| 4 | -154.41 (**-37.85**) | LEV(H5)-LEV(O4) | 1.74 | 157.21 | 0.04 | 0.13 |
| | | HMF(H1)-LEV(O6) | 1.89 | 155.37 | 0.03 | 0.10 |
| | | Water1(Hw)-LEV(O4) | 2.05 | 142.67 | 0.02 | 0.07 |
| 5 | -192.72 (**-38.54**) | LEV(H5)-LEV(O4) | 1.76 | 156.41 | 0.04 | 0.13 |
| | | HMF(H1)-LEV(O6) | 1.9 | 154.61 | 0.03 | 0.10 |
| | | Water1(Hw)-LEV(O4) | 1.81 | 153.2 | 0.03 | 0.12 |
| | | **2HMF:1LEV** | | | | |

| | | | | | | |
|---|---|---|---|---|---|---|
| 0 | Δ*E* = -147.23 kJ $mol^{-1}$ | LEV(H5)-HMF1(O2) | 1.73 | 159.92 | 0.04 | 0.13 |
| | | HMF2(H1)-LEV(O6) | 1.81 | 162.88 | 0.03 | 0.12 |
| | | HMF1(H1)-HMF2(O2) | 1.92 | 148.21 | 0.02 | 0.10 |
| 1 | **-39.43** | LEV(H5)-HMF1(O2) | 1.7 | 162.91 | 0.01 | 0.03 |
| | | HMF2(H1)-LEV(O6) | 1.81 | 162.75 | 0.03 | 0.12 |
| | | HMF1(H1)-HMF2(O2) | 1.78 | 163.83 | 0.04 | 0.12 |
| | | HMF1(H1)-water(Ow) | 1.96 | 166.4 | 0.02 | 0.09 |
| | | Water(Hw)-LEV(O6) | 2.01 | 152.73 | 0.02 | 0.07 |
| 2 | -72.50 (**-36.25**) LEV(H5)-HMF1(O2) become extinct | HMF2(H1)-LEV(O6) | 1.79 | 165.15 | 0.03 | 0.12 |
| | | HMF1(H1)-HMF2(O2) | 1.9 | 150.91 | 0.02 | 0.11 |
| | | Water1(Hw)-HMF1(O3) | 1.74 | 169.72 | 0.04 | 0.13 |
| | | Water2(Hw)-HMF1(O3) | 1.86 | 168.55 | 0.03 | 0.11 |
| | | LEV(H5)-water1(Ow) | 1.71 | 177.12 | 0.04 | 0.13 |
| 3 | -105.62 (**-35.21**) | LEV(H5)-HMF1(O2) | 1.65 | 162.63 | 0.05 | 0.14 |
| | | HMF2(H1)-LEV(O6) | 1.81 | 174.95 | 0.03 | 0.12 |
| | | HMF1(H1)-HMF2(O2) | 1.83 | 171.8 | 0.03 | 0.11 |
| | | Water1(Hw)-LEV(O6) | 1.88 | 157.94 | 0.03 | 0.11 |
| | | Water2(Hw)-HMF2(O3) | 2.28 | 135.79 | 0.01 | 0.04 |
| 4 | -171.20 (**-42.8**) | LEV(H5)-HMF1(O2) | 1.65 | 164.89 | 0.05 | 0.14 |
| | | HMF2(H1)-LEV(O6) | 1.85 | 170.5 | 0.03 | 0.11 |
| | | HMF1(H1)-HMF2(O2) | 1.81 | 165.29 | 0.03 | 0.12 |
| | | Water1(Hw)-LEV(O6) | 1.88 | 172.76 | 0.03 | 0.10 |
| 5 | -193.93 (**-38.79**) | LEV(H5)-HMF1(O2) | 1.63 | 167.85 | 0.05 | 0.14 |
| | | HMF2(H1)-LEV(O6) | 1.84 | 174.20 | 0.03 | 0.11 |
| | | HMF1(H1)-HMF2(O2) | 1.84 | 168.97 | 0.03 | 0.11 |
| | | Water1(Hw)-LEV(O6) | 1.91 | 158.26 | 0.03 | 0.10 |
| | | Water2(Hw)-HMF2(O3) | 2.11 | 134.43 | 0.02 | 0.07 |
| | | **1HMF:1HMF** | | | | |
| 0 | Δ*E* = -78.15 kJ $mol^{-1}$ | HMF1(H1)-HMF2(O2) | 1.86 | 169.29 | 0.03 | 0.10 |
| | | HMF2(H1)-HMF1(O2) | 1.87 | 169.49 | 0.03 | 0.10 |
| 1 | **-38.03** | HMF1(H1)-HMF2(O2) | 1.86 | 169.34 | 0.03 | 0.11 |
| | | HMF2(H1)-HMF1(O2) | 1.79 | 169.95 | 0.03 | 0.12 |
| | | Water(Hw)-HMF2(O3) | 1.81 | 166.25 | 0.03 | 0.12 |
| 2 | -75.05 (**-37.52**) | HMF1(H1)-HMF2(O2) | 1.78 | 173.12 | 0.03 | 0.10 |
| | | HMF2(H1)-HMF1(O2) | 1.86 | 169.79 | 0.03 | 0.12 |
| | | Water1(Hw)-HMF2(O3) | 1.72 | 172.62 | 0.04 | 0.13 |
| 3 | -105.52 (**-35.17**) | HMF1(H1)-HMF2(O2) | 1.82 | 168.32 | 0.03 | 0.11 |
| | | HMF2(H1)-HMF1(O2) | 1.88 | 165.12 | 0.03 | 0.10 |
| | | Water1(Hw)-HMF1(O2) | 1.91 | 151.98 | 0.03 | 0.10 |
| 4 | -160.86 (**-40.21**) | HMF1(H1)-HMF2(O2) | 1.90 | 167.99 | 0.03 | 0.11 |
| | | HMF2(H1)-HMF1(O2) | 1.86 | 167.98 | 0.03 | 0.10 |
| | | Water1(Hw)-HMF2(O2) | 1.90 | 158.32 | 0.03 | 0.10 |
| | | Water2(Hw)-HMF1(O1) | 2.08 | 154.74 | 0.02 | 0.07 |
| 5 | -208.53 (**-41.71**) | HMF1(H1)-HMF2(O2) | 1.88 | 169.53 | 0.03 | 0.10 |

| | | | | | | |
|---|---|---|---|---|---|---|
| | | HMF2(H1)-HMF1(O2) | 1.87 | 169.60 | 0.03 | 0.10 |
| | | Water1(Hw)-HMF1(O2) | 1.98 | 151.71 | 0.02 | 0.08 |
| | | Water2(Hw)-HMF2(O2) | 1.83 | 164.83 | 0.03 | 0.11 |
| **1LEV:1LEV** | | | | | | |
| 0 | Δ*E* = -89.63 kJ mol$^{-1}$ | LEV1(H5)-LEV2(O6) | 1.82 | 173.9 | 0.03 | 0.11 |
| | | LEV2(H6)-LEV2(O4) | 1.73 | 154.95 | 0.04 | 0.13 |
| 1 | **-34.06** | LEV1(H5)-LEV2(O6) | 1.8 | 175.8 | 0.03 | 0.12 |
| | | COOH(LEV2)-O(W1) | 1.67 | 172.22 | 0.05 | 0.14 |
| | | Water(Hw)-LEV2(O4) | 1.76 | 161.13 | 0.04 | 0.13 |
| 2 | -60.49 (**-30.24**) | LEV1(H5)-LEV2(O6) | 1.81 | 173.74 | 0.03 | 0.12 |
| | | LEV2(H6)-LEV2(O4) | 1.72 | 156.96 | 0.04 | 0.13 |
| | | Water1(Hw)-LEV2(O4) | 1.91 | 149.65 | 0.03 | 0.10 |
| 3 | -93.92 (**-31.31**) | LEV1(H5)-LEV2(O6) | 1.79 | 177.53 | 0.03 | 0.12 |
| | | LEV2(H6)-LEV2(O4) | 1.69 | 156.78 | 0.05 | 0.14 |
| | | Water1(Hw)-LEV1(O4) | 1.87 | 171.04 | 0.03 | 0.11 |
| 4 | -128.81 (**-32.20**) | LEV1(H5)-LEV2(O6) | 1.83 | 164.93 | 0.03 | 0.11 |
| | | LEV2(H6)-LEV2(O4) | 1.73 | 155.86 | 0.04 | 0.13 |
| | | Water1(Hw)-LEV2(O4) | 2.2 | 126.4 | 0.02 | 0.06 |
| | | Water(Hw)-LEV1(O6) | 2.13 | 152.17 | 0.02 | 0.09 |
| | | Water3(Hw)-LEV2(O4) | 1.91 | 171.24 | 0.02 | 0.06 |
| 5 | -199.09 (**-39.81**) | LEV1(H5)-LEV2(O6) | 1.81 | 173.67 | 0.03 | 0.12 |
| | | LEV2(H6)-LEV2(O4) | 1.73 | 155.67 | 0.04 | 0.13 |
| | | Water1(Hw)-LEV2(O4) | 2.05 | 142.68 | 0.02 | 0.07 |

**Table S6** Average number of hydrogen bonds, $N_{H\text{-}bonds}$, per number of acceptor molecules for the most relevant hydrogen bonds formed upon HMF:LEV mixtures equilibration from molecular dynamics simulations at the considered HMF:LEV molar ratios and temperature conditions. Donor-acceptor distance and angle of 3.5 Å and 60°, respectively, were used as cutoffs for hydrogen bond definition.

| Hbond (O---H) | Chemical Composition and Temperature | | | | | | | |
|---|---|---|---|---|---|---|---|---|
| | 1HMF:2LEV (283 K) | | 1HMF:2LEV (313 K) | | 1HMF:1LEV (313 K) | | 2HMF:1LEV (313 K) | |
| | Pure | Water | Pure | Water | Pure | Water | Pure | Water |
| HMF(O3)-HMF(H1) | 0.06 | 0.06 | 0.04 | 0.05 | 0.07 | 0.08 | 0.11 | 0.10 |
| HMF(O3)-LEV(H5) | 0.20 | 0.19 | 0.16 | 0.18 | 0.13 | 0.12 | 0.09 | 0.08 |
| Water(Ow)-HMF(H1) | - | 0.26 | - | 0.32 | - | 0.52 | - | 0.07 |
| HMF(O2)-HMF(H1) | 0.14 | 0.10 | 0.18 | 0.13 | 0.21 | 0.19 | 0.31 | 0.29 |
| HMF(O2)-LEV(H5) | 0.25 | 0.25 | 0.29 | 0.31 | 0.22 | 0.20 | 0.14 | 0.15 |
| LEV(O4)-HMF(H1) | 0.18 | 0.14 | 0.15 | 0.13 | 0.27 | 0.22 | 0.34 | 0.29 |
| LEV(O4)-LEV(H5) | 0.36 | 0.29 | 0.37 | 0.32 | 0.28 | 0.26 | 0.22 | 0.18 |
| LEV(O6)-HMF(H1) | 0.09 | 0.12 | 0.12 | 0.16 | 0.16 | 0.17 | 0.20 | 0.21 |
| LEV(O6)-LEV(H5) | 0.21 | 0.20 | 0.20 | 0.16 | 0.17 | 0.15 | 0.11 | 0.08 |
| HMF(O3)-Water(Hw) | - | 0.04 | - | 0.03 | - | 0.04 | - | 0.04 |
| Water(Ow)-Water(Hw) | - | 0.25 | - | 0.18 | - | 0.28 | - | 0.19 |
| HMF(O2)-Water(Hw) | - | 0.07 | - | 0.06 | - | 0.05 | - | 0.05 |
| LEV(O4)-Water(Hw) | - | 0.09 | - | 0.10 | - | 0.08 | - | 0.07 |
| LEV(O6) -Water(Hw) | - | 0.05 | - | 0.07 | - | 0.03 | - | 0.04 |

**Table S7** Domain analysis of HMF, LEV and water molecules from molecular dynamics simulations in HMF:LEV mixtures and HMF:LEV + water mixtures at the considered HMF:LEV molar ratio and temperature conditions.

| HMF:LEV ratio | *T* / K | *wt.* % water | HMF domain count | LEV domain count | Water domain count |
|---|---|---|---|---|---|
| 1:2 | 283 | 0 | 2.97 | 1 | - |
| | | 1.16 | 4.5 | 1 | 38.73 |
| | 313 | 0 | 2.68 | 1 | - |
| | | 1.12 | 1 | 1 | 41.29 |
| 1:1 | 313 | 0 | 1 | 1 | - |
| | | 1.16 | 1 | 1 | 27.48 |
| 2:1 | 313 | 0 | 1 | 1 | - |
| | | 1.22 | 1 | 1 | 28.98 |

Predicted diffusion coefficients (*D* – **Table S8**, and velocity distribution functions (*vdf's*) – **Figure S8**, characterized the system dynamic properties. Calculated HMF self-diffusion values, $D_{HMF}$, are higher than those of LEV molecules, $D_{LEV}$, for all the considered HMF:LEV systems, in agreement with NMR measurements, these values become higher for HMF:LEV + water systems. Highest *D* values correspond to 1HMF:2LEV mixture, thus, unveiling faster diffusion and increased molecular mobility of species for the eutectic composition. *vdf's* of HMF, LEV and water molecules showed roughly the same displacement velocities (900 to 1000 pm × $ps^{-1}$) within the considered fluids. Motion patterns show 1:2 (283 K) < 2:1 (313 K) < 1:1 (313 K) < 1:2 (313 K) trend, in both HMF:LEV and HMF:LEV + water mixtures, thus, discarding water effects. These results, are consistent with self-diffusion coefficients from NMR measurements and MD simulations. Hence,

the analysis of dynamic properties shows enhanced motion behavior for the eutectic mixture driven by the strong HMF-LEV intermolecular interactions abovementioned.

**Table S8** Center of mass self-diffusion coefficients (*D*) experimentally obtained from NMR and calculated from MD simulations for HMF:LEV and HMF:LEV + water mixtures

| | | | $D_{HMF}$ / × $10^{-11}$ $m^2$ × s | | $D_{LEV}$ / × $10^{-11}$ $m^2$ × s | | $D_{H2O}$ / × $10^{-11}$ $m^2$ × s |
|---|---|---|---|---|---|---|---|
| **HMF:LEV molar ratio** | ***T* / K** | ***wt.* % water** | **Exp.** | **MD** | **Exp.** | **MD** | **MD** |
| 1:2 | 283 | 0 | - | 12.11 | - | 8.66 | - |
| | | 1.16 | - | 14.15 | - | 13.54 | - |
| | 313 | 0 | - | 9.72 | - | 9.95 | - |
| | | 1.12 | 7.76 | 13.10 | 7.08 | 12.37 | 32.3 |
| 1:1 | 313 | 0 | - | 7.86 | - | 6.16 | - |
| | | 1.16 | 8.08 | 8.02 | 7.23 | 5.49 | 10.2 |
| 2:1 | 313 | 0 | - | 8.94 | - | 8.64 | - |
| | | 1.22 | 7.17 | 13.98 | 6.72 | 9.50 | 6.84 |

*D*'s obtained from MD simulations where calculated from *msd's* plots in the 6000 to 10000 ps region.

**Table S9** . Experimental and MD-predicted density ($\rho$) for HMF:LEV mixtures

| HMF:LEV ratio | *wt.* % water | *T* /K | $\rho^{MD}$/ g × $cm^{-3}$ | $\rho^{Exp.}$ / g × $cm^{-3}$ |
|---|---|---|---|---|
| 1:2 | 0 | 283 | 1.229 | - |
| | 1.16 | 283 | 1.231 | 1.189 |
| | 0 | 313 | 1.209 | - |
| | 1.16 | 313 | 1.202 (2.91%) | 1.168 |
| 1:1 | 0 | 313 | 1.220 | - |
| | 1.12 | 313 | 1.215 (2.27%) | 1.188 |
| 2:1 | 0 | 313 | 1.229 | - |
| | 1.22 | 313 | 1.217 (0.81%) | 1.208 |

Parenthesized values represent the overestimation % of MD-predicted density with respect to experimental density.

**Table S10** Initial configuration settings for molecular dynamics simulations of (a) 1HMF:2LEV, (b) 1HMF:1 LEV and (c) 2HMF:1LEV systems considering i) pure HMF:LEV and ii) HMF:LEV + water mixtures. *N* stands for the number of molecules of each type, $N_{atoms}$ for the total number of atoms used in each system, and *L* for the initial dimensions of the cubic simulation boxes in the studied temperature and water content range. Color code: blue stands for HMF, red for LEV and green for water.

| HMF:LEV ratio | *N*(HMF) | *N*(LEV) | *N*(water) | $N_{atoms}$ | *T* / K | *L* / Å |
|---|---|---|---|---|---|---|
| 1:2 | 200 | 400 | 0 | 9400 | 283 | 52 × 52 × 52 |
| | | | | 9400 | 313 | 52 × 52 × 52 |
| | 200 | 400 | 56 | 9568 | 283 | 52 × 52 × 52 |
| | | | | 9568 | 313 | 52 × 52 × 52 |
| 1:1 | 300 | 300 | 0 | 9300 | 313 | 52 × 52 × 52 |
| | | | 45 | 9435 | 313 | 52 × 52 × 52 |
| 2:1 | 400 | 200 | 0 | 9200 | 313 | 52 × 52 × 52 |
| | | | 42 | 9326 | 313 | 52 × 52 × 52 |

***i)* Pure HMF:LEV**

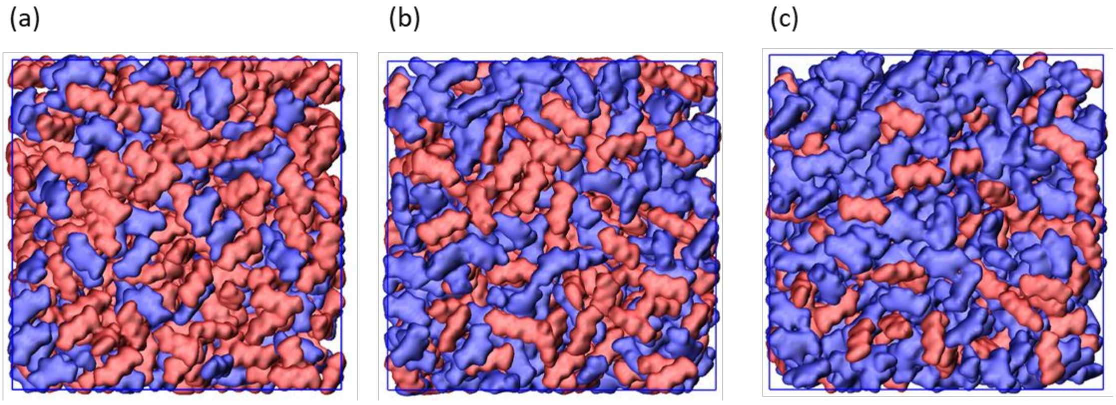


***ii)* HMF:LEV + water**

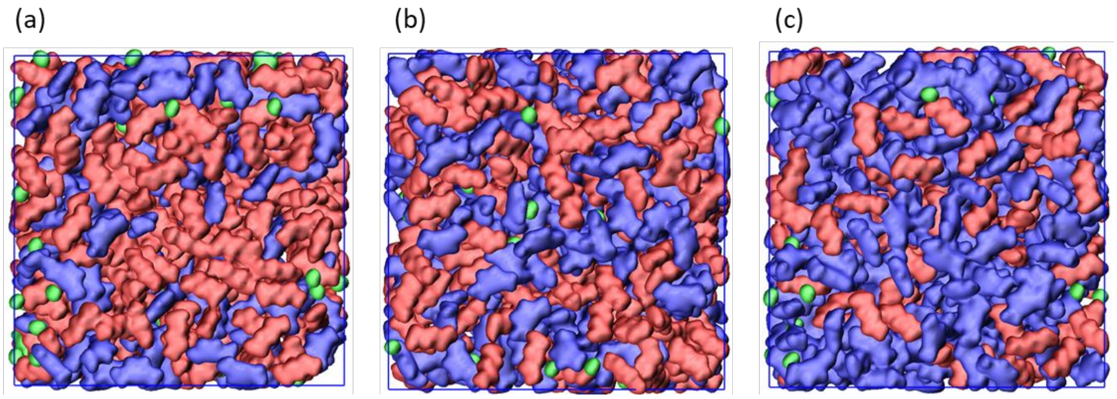


**Table S11** Forcefield parametrization for the compounds studied

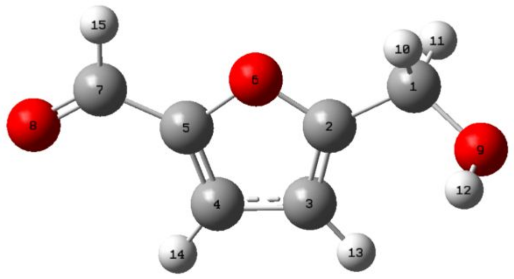

## HMF

| **Label** | ***q*** | ***σ**ᵢᵢ* **/ Å** | ***ε**ᵢᵢ* **/ kJ mol$^{-1}$** | **#** |
|---|---|---|---|---|
| C | 0.312962 | 3.72396 | 0.284512 | 1 |
| C | -0.310048 | 3.72396 | 0.284512 | 2 |
| C | -0.179874 | 3.72396 | 0.284512 | 3 |
| C | 0.161984 | 3.72396 | 0.284512 | 4 |
| C | 0.126568 | 3.58141 | 0.234304 | 5 |
| **O1** | -0.365639 | 3.29632 | 0.8368 | 6 |
| C | 0.383325 | 3.56359 | 0.46024 | 7 |
| **O2** | -0.485761 | 3.02905 | 0.50208 | 8 |
| **O3** | -0.620651 | 3.15378 | 0.636386 | 9 |
| H | 0.059773 | 2.35197 | 0.092048 | 10 |
| H | 0.105572 | 2.35197 | 0.092048 | 11 |
| **H1** | 0.394562 | 0.40001 | 0.192464 | 12 |
| H2 | 0.188062 | 2.35197 | 0.092048 | 13 |
| H3 | 0.187868 | 2.35197 | 0.092048 | 14 |
| H4 | 0.041296 | 2.35197 | 0.092048 | 15 |

**Bonds**

| **Atom Numbers** | | $r_{eq}$ **/Å** | $k_r$**/ kJ mol$^{-1}$ Å$^{-2}$** |
|---|---|---|---|
| 11 | 5 | 1.093 | 1435.0745 |
| 13 | 2 | 1.08 | 1657.8935 |
| 2 | 3 | 1.418 | 1298.672 |

| | | | |
|---|---|---|---|
| 2 | 1 | 1.377 | 2143.279 |
| 14 | 3 | 1.08 | 1657.8935 |
| 3 | 4 | 1.377 | 2143.279 |
| 5 | 1 | 1.471 | 1349.2605 |
| 5 | 9 | 1.418 | 1519.6875 |
| 5 | 10 | 1.093 | 1435.0745 |
| 1 | 6 | 1.36 | 1742.5065 |
| 4 | 6 | 1.36 | 1742.5065 |
| 4 | 7 | 1.423 | 1646.45 |
| 8 | 7 | 1.222 | 3899.333 |
| 7 | 15 | 1.101 | 1400.1465 |
| 9 | 12 | 0.972 | 2346.8265 |

**Angles**

| Atom Numbers | | | $\theta_{eq}$ / deg | $k_\theta$ / kJ mol$^{-1}$ rad$^{-2}$ |
|---|---|---|---|---|
| 2 | 1 | 5 | 131.378 | 443.83 |
| 2 | 1 | 6 | 110.108 | 623.28 |
| 5 | 1 | 6 | 115.253 | 707.6 |
| 1 | 2 | 3 | 108.239 | 521.51 |
| 1 | 2 | 13 | 126.17 | 301.71 |
| 3 | 2 | 13 | 127.405 | 328.8 |
| 2 | 3 | 4 | 108.239 | 521.51 |
| 2 | 3 | 14 | 127.405 | 328.8 |
| 4 | 3 | 14 | 126.17 | 301.71 |
| 3 | 4 | 6 | 110.108 | 623.28 |
| 3 | 4 | 7 | 130.065 | 461.29 |
| 6 | 4 | 7 | 117.219 | 697.36 |
| 1 | 5 | 9 | 106.535 | 813.59 |
| 1 | 5 | 10 | 110.467 | 373.97 |
| 1 | 5 | 11 | 110.467 | 373.97 |
| 9 | 5 | 10 | 108.577 | 470.32 |
| 9 | 5 | 11 | 108.577 | 470.32 |
| 10 | 5 | 11 | 108.836 | 310.74 |
| 1 | 6 | 4 | 106.313 | 766.61 |
| 4 | 7 | 8 | 126.456 | 623.88 |
| 4 | 7 | 15 | 118 | 336.64 |
| 8 | 7 | 15 | 123.439 | 403.48 |
| 5 | 9 | 12 | 106.503 | 477.55 |

**Dihedrals**

| Atom Numbers | | | | $\delta$ / deg | $k_\varphi$ / kJ mol$^{-1}$ | m |
|---|---|---|---|---|---|---|
| 1 | 2 | 3 | 4 | 180 | 14.644 | 2 |
| 1 | 2 | 3 | 14 | 180 | 14.644 | 2 |
| 1 | 5 | 9 | 12 | 0 | 0.4184 | 3 |
| 1 | 6 | 4 | 3 | 180 | 14.644 | 2 |
| 2 | 1 | 6 | 4 | 180 | 14.644 | 2 |
| 2 | 3 | 4 | 6 | 180 | 14.644 | 2 |
| 3 | 2 | 1 | 5 | 180 | 14.644 | 2 |
| 3 | 2 | 1 | 6 | 180 | 14.644 | 2 |
| 3 | 4 | 7 | 8 | 180 | 5.23 | 2 |
| 3 | 4 | 7 | 15 | 180 | 5.23 | 2 |
| 4 | 3 | 2 | 13 | 180 | 14.644 | 2 |

| | | | | | | |
|---|---|---|---|---|---|---|
| 4 | 6 | 1 | 5 | 180 | 14.644 | 2 |
| 5 | 1 | 2 | 13 | 180 | 14.644 | 2 |
| 6 | 1 | 2 | 13 | 180 | 14.644 | 2 |
| 6 | 4 | 3 | 14 | 180 | 14.644 | 2 |
| 6 | 4 | 7 | 8 | 180 | 5.23 | 2 |
| 6 | 4 | 7 | 15 | 180 | 5.23 | 2 |
| 10 | 5 | 9 | 12 | 0 | 1.2468 | 1 |
| 10 | 5 | 9 | 12 | 180 | -0.5774 | 2 |
| 10 | 5 | 9 | 12 | 0 | 0.7238 | 3 |
| 11 | 5 | 9 | 12 | 0 | 1.2468 | 1 |
| 11 | 5 | 9 | 12 | 180 | -0.5774 | 2 |
| 11 | 5 | 9 | 12 | 0 | 0.7238 | 3 |
| 13 | 2 | 3 | 14 | 180 | 14.644 | 2 |

**Improper**

| Atom Numbers | | | | **$\delta$ / deg** | **$k_\varphi$ / kJ mol$^{-1}$** | **m** |
|---|---|---|---|---|---|---|
| 1 | 2 | 6 | 5 | 0 | 30.1081 | 2 |
| 2 | 3 | 1 | 13 | 0 | 3.615 | 2 |
| 3 | 4 | 2 | 14 | 0 | 3.615 | 2 |
| 4 | 6 | 3 | 7 | 0 | 30.1081 | 2 |
| 7 | 8 | 4 | 15 | 0 | 78.291 | 2 |
| 5 | 9 | 1 | 11 | 0 | 0 | 2 |
| 5 | 11 | 1 | 10 | 0 | 0 | 2 |

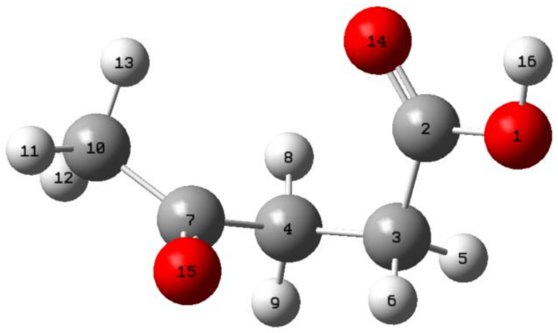


**LEV**

| **Label** | ***q*** | **$\sigma_{ii}$ / Å** | **$\varepsilon_{ii}$ / kJ mol$^{-1}$** | **#** |
|---|---|---|---|---|
| **O5** | -0.618872 | 3.15378 | 0.636386 | 1 |
| C | 0.793998 | 3.56359 | 0.46024 | 2 |
| C | -0.28731 | 3.58141 | 0.234304 | 3 |
| C | -0.144489 | 3.58141 | 0.234304 | 4 |
| H | 0.093689 | 2.35197 | 0.092048 | 5 |
| H | 0.092894 | 2.35197 | 0.092048 | 6 |
| C | 0.764915 | 3.56359 | 0.46024 | 7 |
| H | 0.064584 | 2.35197 | 0.092048 | 8 |
| H | 0.065471 | 2.35197 | 0.092048 | 9 |
| C | -0.677866 | 3.58141 | 0.234304 | 10 |
| H | 0.169694 | 2.35197 | 0.092048 | 11 |
| H | 0.192954 | 2.35197 | 0.092048 | 12 |
| H | 0.171205 | 2.35197 | 0.092048 | 13 |
| **O6** | -0.52399 | 3.02905 | 0.50208 | 14 |
| **O4** | -0.559422 | 3.02905 | 0.50208 | 15 |

| H5 | 0.402545 | 0.40001 | 0.192464 | 16 |
|---|---|---|---|---|

**Bonds**

| Atom Numbers | | $r_{eq}$ /Å | $k_r$/ kJ mol$^{-1}$ Å$^{-2}$ |
|---|---|---|---|
| 13 | 10 | 1.093 | 1435.0745 |
| 8 | 4 | 1.093 | 1435.0745 |
| 6 | 3 | 1.093 | 1435.0745 |
| 14 | 2 | 1.222 | 3899.333 |
| 2 | 1 | 1.355 | 1746.7195 |
| 2 | 3 | 1.492 | 1261.639 |
| 1 | 16 | 0.981 | 2229.093 |
| 4 | 3 | 1.508 | 1282.1115 |
| 4 | 7 | 1.492 | 1261.639 |
| 4 | 9 | 1.093 | 1435.0745 |
| 10 | 7 | 1.492 | 1261.639 |
| 10 | 12 | 1.093 | 1435.0745 |
| 10 | 11 | 1.093 | 1435.0745 |
| 3 | 5 | 1.093 | 1435.0745 |
| 7 | 15 | 1.222 | 3899.333 |

**Angles**

| Atom Numbers | | | $\theta_{eq}$ / deg | $k_\theta$ / kJ mol$^{-1}$ rad$^{-2}$ |
|---|---|---|---|---|
| 2 | 1 | 16 | 111.948 | 351.09 |
| 1 | 2 | 3 | 109.716 | 628.1 |
| 1 | 2 | 14 | 124.425 | 695.55 |
| 3 | 2 | 14 | 124.41 | 564.87 |
| 2 | 3 | 4 | 107.517 | 467.91 |
| 2 | 3 | 5 | 108.385 | 391.44 |
| 2 | 3 | 6 | 108.385 | 391.44 |
| 4 | 3 | 5 | 110.549 | 383 |
| 4 | 3 | 6 | 110.549 | 383 |
| 5 | 3 | 6 | 108.836 | 310.74 |
| 3 | 4 | 7 | 107.517 | 467.91 |
| 3 | 4 | 8 | 110.549 | 383 |
| 3 | 4 | 9 | 110.549 | 383 |
| 7 | 4 | 8 | 108.385 | 391.44 |
| 7 | 4 | 9 | 108.385 | 391.44 |
| 8 | 4 | 9 | 108.836 | 310.74 |
| 4 | 7 | 10 | 118.016 | 693.14 |
| 4 | 7 | 15 | 124.41 | 564.87 |
| 10 | 7 | 15 | 124.41 | 564.87 |
| 7 | 10 | 11 | 108.385 | 391.44 |
| 7 | 10 | 12 | 108.385 | 391.44 |
| 7 | 10 | 13 | 108.385 | 391.44 |
| 11 | 10 | 12 | 108.836 | 310.74 |
| 11 | 10 | 13 | 108.836 | 310.74 |
| 12 | 10 | 13 | 108.836 | 310.74 |

**Dihedrals**

| Atom Numbers | | | | $\delta$ / deg | $k_\varphi$ / kJ mol$^{-1}$ | m |
|---|---|---|---|---|---|---|
| 1 | 2 | 3 | 4 | 0 | -0.2469 | 1 |

| | | | | | | |
|---|---|---|---|---|---|---|
| 1 | 2 | 3 | 4 | 180 | -0.6987 | 2 |
| 1 | 2 | 3 | 4 | 0 | 0.4226 | 3 |
| 1 | 2 | 3 | 5 | 180 | -1.3054 | 2 |
| 1 | 2 | 3 | 5 | 0 | 0.6904 | 3 |
| 1 | 2 | 3 | 6 | 180 | -1.3054 | 2 |
| 1 | 2 | 3 | 6 | 0 | 0.6904 | 3 |
| 2 | 3 | 4 | 7 | 0 | 0.9288 | 1 |
| 2 | 3 | 4 | 7 | 0 | -2.3849 | 3 |
| 2 | 3 | 4 | 8 | 0 | -0.5356 | 1 |
| 2 | 3 | 4 | 8 | 180 | 0.1213 | 2 |
| 2 | 3 | 4 | 9 | 0 | -0.5356 | 1 |
| 2 | 3 | 4 | 9 | 180 | 0.1213 | 2 |
| 3 | 2 | 1 | 16 | 0 | -2.4393 | 1 |
| 3 | 2 | 1 | 16 | 180 | 10.6232 | 2 |
| 3 | 2 | 1 | 16 | 0 | -1.1422 | 3 |
| 3 | 4 | 7 | 10 | 0 | 0.2134 | 1 |
| 3 | 4 | 7 | 10 | 180 | 0.3682 | 2 |
| 3 | 4 | 7 | 10 | 0 | 1.1422 | 3 |
| 3 | 4 | 7 | 15 | 0 | 1.7238 | 1 |
| 3 | 4 | 7 | 15 | 180 | 0.2929 | 2 |
| 3 | 4 | 7 | 15 | 0 | 0.682 | 3 |
| 4 | 3 | 2 | 14 | 0 | 1.7238 | 1 |
| 4 | 3 | 2 | 14 | 180 | 0.2929 | 2 |
| 4 | 3 | 2 | 14 | 0 | 0.682 | 3 |
| 4 | 7 | 10 | 11 | 0 | -0.1506 | 1 |
| 4 | 7 | 10 | 11 | 180 | 0.1799 | 2 |
| 4 | 7 | 10 | 11 | 0 | 1.1129 | 3 |
| 4 | 7 | 10 | 12 | 0 | -0.1506 | 1 |
| 4 | 7 | 10 | 12 | 180 | 0.1799 | 2 |
| 4 | 7 | 10 | 12 | 0 | 1.1129 | 3 |
| 4 | 7 | 10 | 13 | 0 | -0.1506 | 1 |
| 4 | 7 | 10 | 13 | 180 | 0.1799 | 2 |
| 4 | 7 | 10 | 13 | 0 | 1.1129 | 3 |
| 5 | 3 | 2 | 14 | 0 | 1.3807 | 1 |
| 5 | 3 | 2 | 14 | 180 | -2.9455 | 2 |
| 5 | 3 | 2 | 14 | 0 | 0.6443 | 3 |
| 5 | 3 | 4 | 7 | 0 | -0.5356 | 1 |
| 5 | 3 | 4 | 7 | 180 | 0.1213 | 2 |
| 5 | 3 | 4 | 8 | 0 | 0.5941 | 1 |
| 5 | 3 | 4 | 8 | 180 | -2.8995 | 2 |
| 5 | 3 | 4 | 8 | 0 | 0.6569 | 3 |
| 5 | 3 | 4 | 9 | 0 | 0.5941 | 1 |
| 5 | 3 | 4 | 9 | 180 | -2.8995 | 2 |
| 5 | 3 | 4 | 9 | 0 | 0.6569 | 3 |
| 6 | 3 | 2 | 14 | 0 | 1.3807 | 1 |
| 6 | 3 | 2 | 14 | 180 | -2.9455 | 2 |
| 6 | 3 | 2 | 14 | 0 | 0.6443 | 3 |
| 6 | 3 | 4 | 7 | 0 | -0.5356 | 1 |
| 6 | 3 | 4 | 7 | 180 | 0.1213 | 2 |
| 6 | 3 | 4 | 8 | 0 | 0.5941 | 1 |
| 6 | 3 | 4 | 8 | 180 | -2.8995 | 2 |
| 6 | 3 | 4 | 8 | 0 | 0.6569 | 3 |

| | | | | | | |
|---|---|---|---|---|---|---|
| 6 | 3 | 4 | 9 | 0 | 0.5941 | 1 |
| 6 | 3 | 4 | 9 | 180 | -2.8995 | 2 |
| 6 | 3 | 4 | 9 | 0 | 0.6569 | 3 |
| 8 | 4 | 7 | 10 | 0 | -0.1506 | 1 |
| 8 | 4 | 7 | 10 | 180 | 0.1799 | 2 |
| 8 | 4 | 7 | 10 | 0 | 1.1129 | 3 |
| 8 | 4 | 7 | 15 | 0 | 1.3807 | 1 |
| 8 | 4 | 7 | 15 | 180 | -2.9455 | 2 |
| 8 | 4 | 7 | 15 | 0 | 0.6443 | 3 |
| 9 | 4 | 7 | 10 | 0 | -0.1506 | 1 |
| 9 | 4 | 7 | 10 | 180 | 0.1799 | 2 |
| 9 | 4 | 7 | 10 | 0 | 1.1129 | 3 |
| 9 | 4 | 7 | 15 | 0 | 1.3807 | 1 |
| 9 | 4 | 7 | 15 | 180 | -2.9455 | 2 |
| 9 | 4 | 7 | 15 | 0 | 0.6443 | 3 |
| 11 | 10 | 7 | 15 | 0 | 1.3807 | 1 |
| 11 | 10 | 7 | 15 | 180 | -2.9455 | 2 |
| 11 | 10 | 7 | 15 | 0 | 0.6443 | 3 |
| 12 | 10 | 7 | 15 | 0 | 1.3807 | 1 |
| 12 | 10 | 7 | 15 | 180 | -2.9455 | 2 |
| 12 | 10 | 7 | 15 | 0 | 0.6443 | 3 |
| 13 | 10 | 7 | 15 | 0 | 1.3807 | 1 |
| 13 | 10 | 7 | 15 | 180 | -2.9455 | 2 |
| 13 | 10 | 7 | 15 | 0 | 0.6443 | 3 |
| 14 | 2 | 1 | 16 | 0 | 3.4769 | 1 |
| 14 | 2 | 1 | 16 | 180 | 12.87 | 2 |
| 14 | 2 | 1 | 16 | 0 | -0.1213 | 3 |

**Improper**

| Atom Numbers | | | | **$\delta$ / deg** | **$k_\varphi$ / kJ mol$^{-1}$** | **m** |
|---|---|---|---|---|---|---|
| 2 | 3 | 1 | 14 | 0 | 84.9101 | 2 |
| 3 | 4 | 2 | 6 | 0 | 0 | 2 |
| 3 | 6 | 2 | 5 | 0 | 0 | 2 |
| 4 | 7 | 3 | 8 | 0 | 0 | 2 |
| 4 | 8 | 3 | 9 | 0 | 0 | 2 |
| 7 | 15 | 4 | 10 | 0 | 87.9226 | 2 |
| 10 | 13 | 7 | 12 | 0 | 0 | 2 |
| 10 | 13 | 7 | 11 | 0 | 0 | 2 |

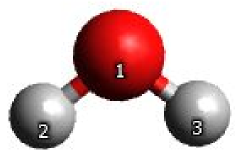


## $H_2O$

| **Label** | ***q*** | **$\sigma_{ii}$ / Å** | **$\varepsilon_{ii}$ / kJ mol$^{-1}$** | **#** |
|---|---|---|---|---|

| | | | | |
|---|---|---|---|---|
| **Ow** | -0.8476 | 3.166 | 0.650194 | 1 |
| **Hw** | 0.4238 | 0 | 0 | 2 |
| **Hw** | 0.4238 | 0 | 0 | 3 |

| Sample | Nitrogen atmosphere | | | | | Air Flow | | | | |
|---|---|---|---|---|---|---|---|---|---|---|
| | $T_{3\%}$ (°C) | $T_{5\%}$ (°C) | $T_{10\%}$ (°C) | $T_{DTGA(max)}$ (°C) | $R_{500°C}$ (%) | $T_{3\%}$ (°C) | $T_{5\%}$ (°C) | $T_{10\%}$ (°C) | $T_{DTGA(max)}$ (°C) | $R_{500°C}$ (%) |
| HMF | 144,7 | 156,7 | 172,5 | 220,5 | 3,1 | 136,3 | 152 | 171,2 | 226 | 4,7 |
| LEV | 112,3 | 122,8 | 137,3 | 192,3 | 0,7 | 113,8 | 146,8 | 164,7 | 226,3 | 0,2 |
| HMF-LEV 1:2 | 143,8 | 158,6 | 177,0 | 230,0 | 8,8 | 132,0 | 149,0 | 169,3 | 224,0 | 8,2 |

**Bonds**

| **Atom Numbers** | | $r_{eq}$ /Å | $k_r$/ kJ mol$^{-1}$ Å$^{-2}$ |
|---|---|---|---|
| 1 | 2 | 1 | 2205.81 |
| 1 | 3 | 1 | 2205.81 |

**Angles**

| **Atom Numbers** | | | $\theta_{eq}$ / deg | $k_\theta$ / kJ mol$^{-1}$ rad$^{-2}$ |
|---|---|---|---|---|
| 2 | 1 | 3 | 109.47 | 158.78 |

**Table S12** Temperatures at 3% ($T_{3\%}$), 5 % ($T_{5\%}$) and 10 % ($T_{10\%}$) mass loss, maximum mass loss derivative temperature ($T_{DTGA(max)}$) and residue at 500 °C ($R_{500°C}$), range of temperature loss and total mass loss of starting materials (HMF and LEV) and of the eutectic mixture (1HMF:2LEV) under $N_2$ atmosphere and air flow

## References


(1) John M. Prausnitz, R. N. L. E. G. de A. *Molecular Thermodynamics of Fluid-Phase Equilibria*, 3rd ed.; Pearson Education, 1998.

(2) Renon, H.; Prausnitz, J. M. Local Compositions in Thermodynamic Excess Functions for Liquid Mixtures. *AIChE Journal* **1968**, *14* (1), 135–144.

(3) Neese, F. Software Update: The ORCA Program System—Version 5.0. *WIREs Computational Molecular Science* **2022**, *12* (5).

(4) Baer, R.; Neuhauser, D. Density Functional Theory with Correct Long-Range Asymptotic Behavior. *Phys Rev Lett* **2005**, *94* (4), 043002.

(5) Becke, A. D. Density-Functional Thermochemistry. III. The Role of Exact Exchange. *J Chem Phys* **1993**, *98* (7), 5648–5652.

(6) Grimme, S.; Antony, J.; Ehrlich, S.; Krieg, H. A Consistent and Accurate Ab Initio Parametrization of Density Functional Dispersion Correction (DFT-D) for the 94 Elements H-Pu. *J Chem Phys* **2010**, *132* (15).

(7) Zhang, J.; Dolg, M. ABCluster: The Artificial Bee Colony Algorithm for Cluster Global Optimization. *Physical Chemistry Chemical Physics* **2015**, *17* (37), 24173–24181.

(8) Bannwarth, C.; Caldeweyher, E.; Ehlert, S.; Hansen, A.; Pracht, P.; Seibert, J.; Spicher, S.; Grimme, S. Extended Tight-binding Quantum Chemistry Methods. *WIREs Computational Molecular Science* **2021**, *11* (2).

(9) Leclercq, J. M.; Allavena, M.; Bouteiller, Y. On the Basis Set Superposition Error in Potential Surface Investigations. I. Hydrogen-Bonded Complexes with Standard Basis Set Functions. *J Chem Phys* **1983**, *78* (7), 4606–4611.

(10) Johnson, E. R.; Keinan, S.; Mori-Sánchez, P.; Contreras-García, J.; Cohen, A. J.; Yang, W. Revealing Noncovalent Interactions. *J Am Chem Soc* **2010**, *132* (18), 6498–6506.

(11) Klamt, A. Conductor-like Screening Model for Real Solvents: A New Approach to the Quantitative Calculation of Solvation Phenomena. *J Phys Chem* **1995**, *99* (7), 2224–2235.

(12) Acree, W.; Chickos, J. S. Phase Transition Enthalpy Measurements of Organic and Organometallic Compounds. Sublimation, Vaporization and Fusion Enthalpies From 1880 to 2010. *J Phys Chem Ref Data* **2010**, *39* (4).

(13) Lyubartsev, A. P.; Laaksonen, A. M.DynaMix – a Scalable Portable Parallel MD Simulation Package for Arbitrary Molecular Mixtures. *Comput Phys Commun* **2000**, *128* (3), 565–589.

(14) Halgren, T. A. Merck Molecular Force Field. I. Basis, Form, Scope, Parameterization, and Performance of MMFF94. *J Comput Chem* **1996**, *17* (5–6), 490–519.

(15) Zoete, V.; Cuendet, M. A.; Grosdidier, A.; Michielin, O. SwissParam: A Fast Force Field Generation Tool for Small Organic Molecules. *J Comput Chem* **2011**, *32* (11), 2359–2368.

(16) Breneman, C. M.; Wiberg, K. B. Determining Atom-centered Monopoles from Molecular Electrostatic Potentials. The Need for High Sampling Density in Formamide Conformational Analysis. *J Comput Chem* **1990**, *11* (3), 361–373.

(17) Martínez, L.; Andrade, R.; Birgin, E. G.; Martínez, J. M. PACKMOL: A Package for Building Initial Configurations for Molecular Dynamics Simulations. *J Comput Chem* **2009**, *30* (13), 2157–2164.

(18) Tuckerman, M.; Berne, B. J.; Martyna, G. J. Reversible Multiple Time Scale Molecular Dynamics. *J Chem Phys* **1992**, *97* (3), 1990–2001.

(19) Essmann, U.; Perera, L.; Berkowitz, M. L.; Darden, T.; Lee, H.; Pedersen, L. G. A Smooth Particle Mesh Ewald Method. *J Chem Phys* **1995**, *103* (19), 8577–8593.

(20) Brehm, M.; Kirchner, B. TRAVIS - A Free Analyzer and Visualizer for Monte Carlo and Molecular Dynamics Trajectories. *J Chem Inf Model* **2011**, *51* (8), 2007–2023.

(21) Humphrey, W.; Dalke, A.; Schulten, K. VMD: Visual Molecular Dynamics. *J Mol Graph* **1996**, *14* (1), 33–38.

(22) Stejskal, E. O.; Tanner, J. E. Spin Diffusion Measurements: Spin Echoes in the Presence of a Time-Dependent Field Gradient. *J Chem Phys* **1965**, *42* (1), 288–292.

(23) Hayamizu, K.; Tsuzuki, S.; Seki, S.; Umebayashi, Y. Nuclear Magnetic Resonance Studies on the Rotational and Translational Motions of Ionic Liquids Composed of 1-Ethyl-3-Methylimidazolium Cation and Bis(Trifluoromethanesulfonyl)Amide and Bis(Fluorosulfonyl)Amide Anions and Their Binary Systems Including Lithium Salts. *J Chem Phys* **2011**, *135* (8).

(24) Wales, D. J.; Walsh, T. R. Theoretical Study of the Water Pentamer. *J Chem Phys* **1996**, *105* (16), 6957–6971
